# Potential of conserved antigenic sites in development of universal SARS-like coronavirus vaccines

**DOI:** 10.3389/fimmu.2022.952650

**Published:** 2022-09-20

**Authors:** Siling Wang, Dinghui Wu, Hualong Xiong, Juan Wang, Zimin Tang, Zihao Chen, Yizhen Wang, Yali Zhang, Dong Ying, Xue Lin, Chang Liu, Shaoqi Guo, Weikun Tian, Yajie Lin, Xiaoping Zhang, Quan Yuan, Hai Yu, Tianying Zhang, Zizheng Zheng, Ningshao Xia

**Affiliations:** ^1^ State Key Laboratory of Molecular Vaccinology and Molecular Diagnostics, National Institute of Diagnostics and Vaccine Development in Infectious Diseases, School of Life Sciences, School of Public Health, Xiamen University, Xiamen, China; ^2^ Department of Pulmonary Medicine, The First Affiliated Hospital of Xiamen University, Xiamen, China

**Keywords:** SARS-CoV-2, COVID-19, convalescent individual, conserved antigenic sites, cross-neutralizing antibodies, universal vaccine

## Abstract

Given pandemic risks of zoonotic SARS-CoV-2 variants and other SARS-like coronaviruses in the future, it is valuable to perform studies on conserved antigenic sites to design universal SARS-like coronavirus vaccines. By using antibodies obtained from convalescent COVID-19 patients, we succeeded in functional comparison of conserved antigenic sites at multiple aspects with each other, and even with SARS-CoV-2 unique antigenic sites, which promotes the cognition of process of humoral immune response to the conserved antigenic sites. The conserved antigenic sites between SARS-CoV-2 and SARS-CoV can effectively induce affinity maturation of cross-binding antibodies, finally resulting in broadly neutralizing antibodies against multiple variants of concern, which provides an important basis for universal vaccine design, however they are subdominant, putatively due to their lower accessibility relative to SARS-CoV-2 unique antigenic sites. Furthermore, we preliminarily design RBDs to improve the immunogenicity of these conserved antigenic sites. Our study focusing on conserved antigenic sites provides insights for promoting the development of universal SARS-like coronavirus vaccines, thereby enhancing our pandemic preparedness.

## Introduction

Severe acute respiratory syndrome coronavirus 2 (SARS-CoV-2) is the cause of the ongoing outbreak of coronavirus disease 2019 (COVID-19), resulting in a global pandemic (1, 2). As of March 14, 2022, SARS-CoV-2 has caused more than 450 million human infections around the world, including approximately 6 million deaths, which has led to unprecedented enormous global health and economic damage. The ~30 kb RNA genome of SARS-CoV-2 encodes four structural proteins including the spike (S), membrane (M), envelope (E) and nucleocapsid (N) proteins, nonstructural proteins, and a number of accessory proteins (3). The transmembrane S glycoprotein is divided into S1, comprising a receptor-binding domain (RBD) and an N-terminal domain (NTD), and S2 promoting membrane fusion *via* a fusion peptide. As most neutralizing monoclonal antibodies (mAbs) isolated from convalescent COVID-19 patients target the RBD (4–8), by which the S protein binds to receptor angiotensin-converting enzyme 2 (ACE2) and promotes the exposure of the fusion peptides within the S2 component to contribute to viral membrane fusion with host cells, this domain is the main target for the design of therapeutics and vaccines (9, 10).

Human coronaviruses (HCoVs) include HCoV-OC43, HCoV-HKU1, HCoV-229E and HCoV-NL63, as well as highly pathogenic Middle East respiratory syndrome coronavirus (MERS-CoV), SARS-CoV and SARS-CoV-2. SARS-CoV-2, which is phylogenetically close to SARS-CoV, is classified in the *Betacoronavirus* genus, which includes another highly pathogenic virus, MERS-CoV, as well as HCoV-OC43 and HCoV-HKU1 variants leading to endemic disease (11, 12). Historically, there have been three HCoV infection outbreaks causing a severe syndrome, including the SARS outbreak that was initially identified as an exotic infection in coronavirus evolution, the MERS-CoV outbreak that was the second most severe outbreak, and the current COVID-19 pandemic (13–16). Studies reveal that it is reasonable to speculate on the possibility of emergence of other SARS-like coronaviruses in the future. Hence, it is certainly valuable to promote the development of more universal coronavirus vaccines and broader therapeutic agents by characterizing conserved antigenic sites in SARS-CoV-2 RBD to enhance our preparedness against the possible pandemic risk of SARS-like coronaviruses in the future. Moreover, as the duration of the SARS-CoV-2 pandemic extends, multiple variants of concern (VOCs) have emerged around the world. Although the D614G mutation in the S protein significantly promotes corresponding variant infectivity in susceptible cells, this residue substitution fails to cause immune escape (17–21). Conversely, multiple studies support that the Beta, Gamma, Delta and Omicron variants could decrease the therapeutic efficacy of neutralizing antibodies (NAbs) and even compromise the protective efficacy of approved SARS-CoV-2 vaccines targeting the initial SARS-CoV-2 strain that emerged in 2019 (20, 22–26). Hence, universal SARS-like coronavirus vaccines based on the conserved antigenic sites in the RBD also have potential in preventing highly pathogenic SARS-CoV-2 variants that could escape established specific immune memory.

Although SARS-CoV-2 and SARS-CoV share 90% amino acid identity in the S2 domain, the SARS-CoV-2 RBD shows only 73% amino acid identity with the SARS-CoV RBD (12), implying that there may be fewer conserved antigenic sites within the RBD. Nevertheless, some highly conserved epitopes in the SARS-CoV RBD have been identified by the mAbs CR3022 (27), S309 (28) and ADI-56046 (6), cross-binding mAbs that were originally isolated from SARS patients, among which S309 and ADI-56046 could efficiently neutralize infection by SARS-CoV-2 and SARS-CoV. In addition, many human mAbs targeting the SARS-CoV-2 S protein isolated from convalescent COVID-19 patients have been reported; however, cross-binding mAbs, especially cross-neutralizing mAbs, are rarely reported (29–31), indicating that the conserved antigenic sites within the RBD may be subdominant compared to the unique sites. While some conserved antigenic sites have been identified by cross-binding mAbs, including CR3022 (27), S309 (28) and ADI-56046 (6) from SARS-CoV infection survivors and COVA1-16 (5), EY6A (8) and 2-36 (32) from COVID-19 patients, no studies have been performed to quantitatively define an antigenic map of conserved sites in the SARS-CoV-2 RBD.

In this study, 77 SARS-CoV-2 RBD-specific antibodies were isolated from a cohort of 10 convalescent COVID-19 patients for further biophysical characterization, by which we succeeded in defining a quantitative antigenic map of neutralizing sites within the SARS-CoV-2 RBD. We identified multiple conserved antigenic sites with weaker immunogenicity, due to their inaccessibility. To improve immune response to conserved antigenic sites, we tried to design RBDs, which might contribute to the development of universal SARS-like coronavirus vaccines.

## Materials and methods

### Collection of blood samples

In this study, a total of 10 convalescent COVID-19 individuals infected with SARS-CoV-2 were enrolled, and the peripheral bloods were collected. The study was approved by the institutional review board of the School of Public Health in accordance with the Declaration of Helsinki, and written informed consent was obtained.

### Detection of plasma antibody titer against SARS-CoV-2 RBD

To detect the plasma titers of total antibodies (Ab), IgG and IgM against SARS-CoV-2 RBD, we performed commercial enzyme-linked immunosorbent assay (ELISA) kits (Beijing Wantai Biological Pharmacy Enterprise), according to the manufacturer’s instructions. The Ab-ELISA kit is based recombinant viral antigen using a double-sandwich reaction form. The IgG-ELISA kit is an indirect ELISA assay, and the IgM-ELISA kit is based on μ-chain capture method. The samples were initially tested undiluted, and the positive samples with the signal to a cutoff ratio (S/CO) >=10 were further diluted (1:10, 1:100, 1:1,000 and 1:10,000) by PBS buffer containing 20% newborn bovine serum (NBS) and tested again. The titers for Ab, IgG and IgM antibody were calculated *via* S/CO multiplied by the maximum dilution factors.

### Recombinant expression and purification of SARS-CoV S protein, SARS-CoV-2 S protein and diverse SARS-CoV-2 RBD protein

For SARS-CoV and SARS-CoV-2 S protein, a gene encoding the ectodomain of a prefusion conformation-stabilized S protein was synthesized, composed of SARS-CoV gene sequence (GenBank: ABF65836) or SARS-COV-2 gene sequence (GenBank: MN908947), a C-terminal T4 fibritin trimerization motif, an HRV3C protease and 8xHisTag. To determine the blocking capacity of mAbs, we also synthesized gene of SARS-CoV-2 S fluorescin probe comprising SARS-COV-2 gene sequence, a C-terminal T4 fibritin trimerization motif, an HRV3C protease, 8xHisTag and a C-terminal green fluorescent protein (mGamillus). Moreover, to express mutate, wildtype SARS-CoV-2 RBD, residues 319-518 fused to mouse IgG1 Fc domain. For mutate RBDs, different selected amino acid of RBD were substituted by alanine or arginine on purpose (**Table S3**). Recombinant expressions of these proteins were performed by the ExpiCHO™ expression system (Thermo Scientific, A29133). Briefly, plasmids encoding targeted proteins were transiently transfected into ExpiCHO cells by using ExpiFectamine™ CHO transfection kit (Thermo Scientific, A29129). The cell-free supernatants were obtained 7 days after transfection by centrifugation and filtration with a 0.22 μm filter. Subsequently, the S-related proteins (SARS-CoV S, SARS-CoV-2 S and SARS-CoV-2 S fluorescin probe) were purified by Ni Sepharose Excel resin, and RBD fused to mouse IgG1 Fc domain by Protein A column, and the S and RBD proteins were stored in the PBS buffer.

### Specific memory B cell response and single B cell sorting

RBD specific B cells were obtained in the same way as previously reported. PBMCs collected from 10 individuals were incubated with a cocktail containing live/dead-Aqua, CD3-PE-Cy7, CD19-BV786, CD27-BV650, anti-human IgM-PerCP-Cy5.5, anti-human IgG-BV421, RBD-FITC and biotinylated RBD, followed with Streptavidin-APC binding to biotinylated RBD. The RBD-specific memory B cells were identified as live+CD19+CD3-CD27+IgG+RBD+, and then the single specific memory B cells were sorted by fluorescence activated cell sorting on an Aria III sorter (BD Biosciences) into 96-well PCR plates containing 20 μL per well of lysis buffer [5 μL of 5×first strand buffer (Invitrogen), 1.25 μL dithiothreitol (Invitrogen), 0.5 μL RNase Out (Invitrogen), 0.0625 μL Igepal (Sigma)]. Plates were stored at -80°C prior to reverse transcription reaction.

### Single B cell PCR, cloning and expression of antibody

Antibody variable genes (IgH, Igλ and Igκ) were amplified by RT-PCR and nested PCR reactions as previously described (33). The paired heavy and light chains were then cloned into expression vectors containing the constant regions of human IgG1 and light chain. The paired heavy and light chain expression cassettes were then transiently co-transfected into ExpiCHO cells with equal amounts of plasmids according to the manufacturer’s instructions (Life Technologies), and antibodies were purified from culture supernatant 5-7 days after transfection, using a recombinant protein-A column (GE Healthcare).

### Antibody germline usage and phylogenetic analysis

Antibody gene repertoire was analyzed for the variable region of IgG heavy and light chains using the IMGT V-quest webserver (http://www.imgt.org/IMGT/vquest). Phylogenetic analysis of antibody gene was performed by ggtree R package (34).

### Binding activity assay for mAbs by indirect ELISA

The binding activity of the mAbs against SARS-CoV-2 S protein were determined using an indirect ELISA. The mAbs were added to antigen-coated microwell plates, and incubated at 37°C for 30 min. Then, incubation of HRP-conjugated anti-human antibody at 37°C for 30 min to detect the bound mAbs, followed by washing five times. Finally, substrate solution was added to the wells for 15 min at 37°C, and reaction was stopped by adding 50 μL of 2 M H_2_SO_4_. The optical density (OD) was measured at 450 nm with a reference wavelength of 630 nm. In addition, binding activity of the mAbs against SARS-CoV-2 RBD and SARS-CoV S protein were determined by same method.

### Identification of SARS-CoV-2 RBD critical residues recognized by mAbs

To determine the critical residues, mAbs were conjugated with horse radish peroxidase (HRP). Microwell plates were pre-coated with mutate SARS-CoV-2 RBD and wildtype RBD at 100 ng per well. mAbs-HRP were performed two times gradient dilution with 4 μg/mL begin and incubated at 37°C for 30 min followed by washing five times. Substrate solution was incubated for 15 min at 37°C and stopped by 50 μL of 2 M H_2_SO_4_. OD was determined at 450 nm with a reference wavelength of 630 nm. The binding activity of mAbs against wildtype and mutated RBD was calculated by area under the curve (AUC), and the influence of residues was assessed by binding activity reduction against corresponding mutate SARS-CoV-2 RBD. Residues reducing binding activity by more than 75% are identified as critical residues.

### Blocking capacity of mAbs against SARS-CoV-2 S protein

For SARS-CoV-2 S protein-blocking assay, mAbs were pre-made as 2-fold serial dilutions using DMEM containing 10% FBS. Aliquots (44 μL per well) of diluted samples and S protein probes (11 μL per well) were mixed in a 96-well plate with U shaped bottom. Half of the culture medium (50 μL) of 293T-ACE2iRb3 cell plate were gently removed, and 50 μL of sample/probe mixtures were added to each well. Cell image acquisitions performed with Opera Phenix (green, red and near-infrared channels in confocal mode) using a 20x water immersion objective at 1-hour after probe incubation in wash-free and live-cell conditions.

All quantitative image analyses were based on images that acquired by Opera Phenix. All image data were transfer to Columbus system (version 2.5.0, PerkinElmer Inc) for analysis. Multiparametric image analysis was performed as described in the following. The signals of blue channel or near-infrared channel were used to detect the nucleus. As the ACE2 is a membrane protein, the signals of ACE2-mRuby3 (red channel) were used to determine the cell boundary. Then, the cells were further segment into the regions of membrane (outer border: 0%, inner border: 15%), cytoplasm (outer border: 20%, inner border: 45%), and nucleus (outer border: 55%, inner border: 100%). The MFI of probe channel (Ex488/Em525) in the cytoplasmic region (cMFI). The MFI of ACE2-mRuby3 (Ex561/Em590) on the membrane were also calculated for inter-well normalization. The cMFI inhibition ratio (%) of the test sample was calculated using the following equation: [(cMFIpc-cMFItst)/(cMFIpc-cMFIblk)]×100%. In this formula, the cMFIpc is the cMFI value of probe-only well (as positive control), the cMFItst is the cMFI value of test well and the cMFIblk is the cMFI value of cell-only well. For each plate, five replicates of probe-only well and one cell-only well were included. The blocking capacity of mAbs were expressed as IC50.

### Neutralization capacity of mAbs determined by SARS-CoV and SARS-CoV-2 pseudovirus

SARS-CoV-2 pseudovirus using VSV carrying the SARS-CoV-2 spike protein were produced according to our previous study. Briefly, SARS-CoV-2 S gene was codon optimized for expression in human cells and truncated with 18 amino acids at the C-terminal, then was cloned into the eukaryotic expression vector pCAG to obtain pCAG-nCoVSde18. The plasmid pCAG-nCoVSde18 was transfected into Vero-E6. VSVdG-EGFP-G (Addgene, 31842) virus was inoculated into cells expressing SARS-CoV-2 Sde18 truncated protein and incubated for 1 hour. Then the VSVdG-EGFP-G virus was removed from the supernatant and anti-VSV-G rat serum was added to block the remaining VSVdG-EGFP-G infection. The progeny virus will carry SARS-CoV-2 Sde18 truncated protein. After VSVdG-EGFP-G infection, supernatant was collected, centrifuged and filtered (Millipore, SLHP033RB) to obtain the SARS-CoV-2 pseudovirus without debris. SARS-CoV pseudovirus was constructed by the same method. Finally, pseudovirus was stored for use at -80°C.

To determine the neutralizing capacity, mAbs with 2-fold serial dilutions with 10% FBS-DMEM from 2 μg/mL were mixed with diluted SARS-CoV or SARS-CoV-2 pseudovirus (MOI = 0.05), incubated at 37°C for 1 hour. A mixture of 80 μL was added to the precoated BHK21-hACE2 cells. After incubation for 12 hours, post-infection cells were fluorescently imaged using Opera phenix or Operetta CLS (Perkinelmer), and quantitatively analyzed by Columbus image management analysis software to detect the number of green, fluorescent positive cells. The inhibition rate was calculated by reduction of GFP positive cells with presence of mAbs compared with the untreated control wells.

### Competition binding assay for neutralizing antibodies by ELISA and cluster analysis

Briefly, the unlabeled mAbs (50 μg per well) or PBS were added to RBD-coated 96-well microplates and then incubated for 30 min at 37°C. Next, HRP-conjugated mAbs were added at selected dilutions, at which OD readings was ~1.5 with PBS present. After incubation for 30 min at 37°C, the microplates were rinsed, and the color was developed. The competitive ability was measured quantitatively by comparing OD in the presence and absence of competitor mAbs and transformed using the formula log_2_ (OD_inhibited_/OD_original_). For mAbs to be clustered by competitive ability, clustering distance was calculated by Euclidean, and mAbs were clustered by ward. D2 method using pheatmap R package (version: 1.0.12).

### Mouse immunization

For antibody response evaluation of RBD_Glycan420,475_, RBD_Glycan458,475_, RBD_Trucation455-491_ and RBD_Trucation470-491_, BALB/c mice were immunized with various RBD proteins at 20 μg/dose with FH002C through intramuscular injection. Serum samples were collected at Week 0 and 2 *via* retro-orbital bleeding to measure the antibody titers.

### Measurement of mouse sera IgG titer against SARS-CoV and SARS-CoV-2 S protein

Microplates were pre-coated with recombinant antigens of SARS-CoV or SARS-CoV-2 S protein. For detections, serial-diluted (2-fold) serum samples (100 μL per well) were added into the wells, and the plates were incubated at 37°C for 30 min, followed by washing with PBST buffer (20 mM PB7.4, 150 mM NaCl and 0.05% Tween 20). Then, HRP-conjugated anti-mouse IgG solutions (100 μL per well) were added. After a further 30 min incubation followed by washing, TMB chromogen solution (100 μL per well) was added into the well. 15 min later, the chromogen reaction was stopped by adding 50 μL of 2 M H_2_SO_4_, and the OD450-630 was measured. The IgG titer of each serum was defined as the dilution limit to achieve a positive result (>median+3×SD of ODs of negative controls).

### Statistical analysis

To compare continuous variables, the Mann-Whitney U test and non-paired t test were performed. Linear regression model and Spearman test were used for correlation analyses. For difference analysis, p values less than 0.05 are considered statistically significant. GraphPad Prism (version 8.0.1) was used for all statistical calculations. Analysis of protein structure was performed by PyMOL Molecular Graphics System (version 2.3.0).

## Results

### Seroconversion of antibodies against SARS-CoV-2 in convalescent COVID-19 patients and isolation of SARS-CoV-2 RBD-specific antibodies

We collected blood samples from 10 convalescent COVID-19 patients (**Table S1**). The humoral immune response was efficiently elicited in these convalescent individuals, as indicated by high plasma titers of RBD-specific IgG with differences of less than an order of magnitude (**Table S2**). Since the plasma neutralizing capacity strongly correlates with RBD-specific total antibody and IgG titers, indicating that the RBD of the S protein is the dominant target of nAbs elicited by SARS-CoV-2 infection (**Figure S1**). The proportion of RBD-specific B cells in memory B cells ranged from 0.03% to 0.18%, and RBD-specific memory B cells contained a higher percentage of the IgG subtype than the IgM subtype, revealing that SARS-CoV-2 infection efficiently promoted B cell receptor (BCR) class switching and affinity maturation in convalescent individuals, thus eliciting strong humoral immune response against SARS-CoV-2 (**Figures S2A–C**). Then, 77 RBD-specific monoclonal antibodies (mAbs) were obtained from the 10 convalescent individuals for comprehensive feature description (**Figure S2D**).

### Characterization of SARS-CoV-2 RBD-specific mAbs obtained from convalescent COVID-19 patients

**Figure 1:**
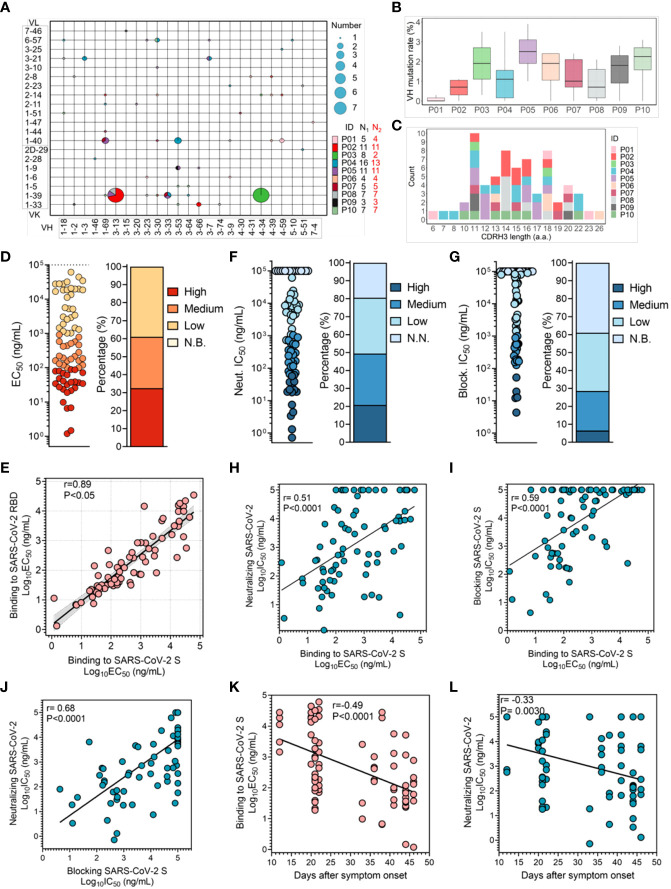
Characterization of SARS-CoV-2 RBD-specific mAbs obtained from convalescent COVID-19 patients. **(A)** V gene frequencies for the heavy and light chains of SARS-CoV-2 RBD-specific antibodies. The size corresponding to the number of heavy and light chain pairs in the repertoires is also denoted. Color indicates different convalescent individuals. N_1_ indicates the number of SARS-CoV-2 RBD-specific antibodies for different individuals, and N_2_ indicates the number of SARS-CoV-2 RBD-specific antibodies with unique clonotypes for different individuals. **(B)** The V region SHM of the heavy chain of specific antibodies from different individuals (N=67). **(C)** Distribution of the CDR3 length of the heavy chain. Antibodies are colored by each individual (N=67). V region germline genes and SHM and CDR3 length were determined using immunogenetics (IMGT). **(D)** The binding activity of specific antibodies to the SARS-CoV-2 S protein was determined by ELISA. Color corresponds to binding activity; high (EC_50_ <100 ng/mL), medium (EC_50_ between 100 ng/mL and 1 μg/mL), low (EC_50_ between 1 μg/mL and 100 μg/mL) and N.B. (EC_50_ >100 μg/mL). **(E)** Correlation between the binding capacities to the SARS-CoV-2 RBD and S protein (N=77). The 95% confidence interval of the regression line is shown in light gray, and the r and P values of the correlation are also indicated. **(F, G)** Neutralizing capacity was determined with a SARS-CoV-2 pseudovirus, and blocking capacity was determined by an S protein binding model. IC50 values are shown in the left panel, and the percentage of mAbs within the indicated IC_50_ range is shown in the right panel. Color represents different neutralizing and blocking capacities; high (IC_50_ <100 ng/mL), medium (IC_50_ between 100 ng/mL and 1 μg/mL), low (IC_50_ between 1 μg/mL and 100 μg/mL), and N.N. or N.B. (IC_50_ >100 μg/mL). **(H, I)** The correlation between binding activity and neutralization or blocking capacity. For the blocking assay, mAbs were pre-made as 2-fold serial dilutions and incubated with S protein probe. The mixture was input into each well coated with 293T-ACE2iRb3 cell. After 1-hour S probe incubation, cell image acquisitions were performed with Opera Phenix. The r and P values of the correlation are also indicated. **(J)** The correlation between neutralization and blocking capacities. The r and P values of the correlation are indicated. **(K)** Correlation between the binding activity of specific antibodies and duration of the immune response in convalescent individuals (N=77). **(L)** The change in neutralization potency of specific mAbs over days after symptom onset. The r and P values of the correlation are indicated. All correlation analyses were performed using the Spearman test.

To characterize SARS-CoV-2 RBD-specific mAb repertoire usage, we compared sequences with the well-defined naïve repertoire of the IMGT database to obtain the assigned germline V region. Based on the exclusion of clonal expansion in P01, P03 and P04, 67 unique clonotypes were identified. Notably, 7 out of 8 antibody sequences obtained from P03 were highly conserved, except for P03-3B1, illustrating that this BCR clonotype was the immunodominant clone in P03 induced by SARS-CoV-2 infection (**Figure 1A** and **Figure S3**). Furthermore, enrichment of multiple VH and VK/VL sequences was observed. In addition, VK1-39-derived light chains were most often combined with the heavy chain of various types of VHs to form antibodies, accounting for 22.4% (15/67) (**Figure S4** and **Figure 1A**). The mean somatic hypermutation (SHM) rate of the heavy chain V region was similar among individuals, and the mean levels (2%) were comparable to those detected in the context of infections with other respiratory viruses (**Figure 1B**) (35–37). Additionally, even though the average length of CDRH3 of RBD-specific mAbs was consistent with that of the naïve repertoire (~15 amino acids), we observed significant enrichment of shorter CDRH3 sequences (11 amino acids) in VH3-53/66-derived mAbs, differing from influenza virus and human immunodeficiency virus (HIV)-1 mAbs (**Figure 1C** and **Figure S8A**) (38–40).

Subsequently, we assessed the binding activity of these mAbs to recombinant S protein and RBD protein fragment of SARS-CoV-2 using enzyme-linked immunosorbent assay (ELISA). mAbs presented diverse binding activity to SARS-CoV-2 S protein, of which 61.0% showed strong binding activity (EC_50_< 1 μg/mL); this result suggested that infection with SARS-CoV-2 can effectively stimulate humoral immune response to produce a large number of specific high-affinity mAbs (**Figure S2A** and **Figure 1D**). Interestingly, while there was a correlation between binding activity to SARS-CoV-2 RBD and to S protein, some mAbs bound more strongly to the RBD, implying that their target epitopes were poorly presented in S protein to be recognized, due to coverage by another RBD monomer or the NTD domain (**Figure 1E**) (41). Next, we assessed the neutralizing activity of these mAbs using a vesicular stomatitis virus (VSV) pseudovirus model carrying the SARS-CoV-2 S protein, and the neutralization IC_50_ potencies are shown in **Figure S5C**. In total, 80.5% (62/77) of these mAbs displayed neutralization of SARS-CoV-2, characterized as nAbs, among which 16 were identified as potent neutralizers with an IC_50_< 0.1 μg/mL, 22 as moderate neutralizers with an IC_50_ of 0.1-1 μg/mL, and 24 as weak neutralizers with an IC_50_ of 1-100 μg/mL (**Figure 1F**). Surprisingly, none of the antibodies isolated from the convalescent individual P03 had a potent neutralizing capacity, although clonal expansion was efficiently elicited (**Figure S6**).

Next, we investigate whether blocking S protein binding to ACE2 is the key neutralizing mechanism. The results showed that 61.0% of the specific mAbs had the ability to block entrance of the S protein into cells, with IC_50_ values ranging from 4 ng/mL to 100 μg/mL (**Figure S5D** and **Figure 1G**). These blocking and neutralizing capacities were well correlated with the binding capacity (**Figures 1H, I**). In terms of the neutralization and blocking data, we found a good correlation, indicating that blocking the attachment of the SARS-CoV-2 S protein to receptor ACE2 was critical for the inhibition of SARS-CoV-2 infection by nAbs targeting RBD (**Figure 1J** and **Figure S7**). However, some nAbs neutralized SARS-CoV-2 with weak blockade of SARS-CoV-2 S protein binding to ACE2 (28). Although the corresponding neutralizing mechanism has not been explained clearly, it is putative that binding of these nAbs may impede sequential conformational changes in the S protein.

To determine whether the affinity of SARS-CoV-2 RBD-specific mAbs efficiently evolves, we analyzed the association between the binding activity of mAbs and the duration of the immune response. The binding activity and neutralizing capacity of specific mAbs correlated with days after symptom onset, illustrating that the affinity of these specific mAbs can continuously evolve (**Figures 1K, L** and **Figure S9**). However, the plasma anti-RBD IgG titer did not correlate with days after symptom onset for individuals, which might resulted from the limitation of the plasma samples (**Figure S1A**) (42). Taken together, humoral immune response is efficiently elicited by SARS-CoV-2 infection, and affinity of functional mAbs is constantly evolving.

### Profile of cross-binding antibodies between SARS-CoV and SARS-CoV-2

**Figure 2:**
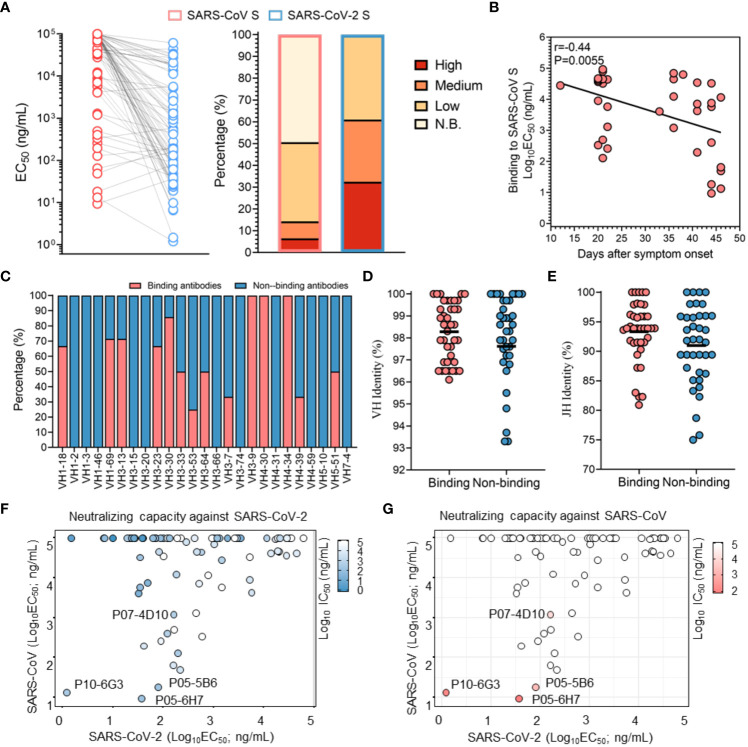
Profile of cross-binding SARS-CoV-2 RBD-specific mAbs. **(A)** The binding activity of antibodies specific for the SARS-CoV S protein and SARS-CoV-2 S protein were determined by ELISA. Color corresponds to binding activity; high (EC_50_ <100 ng/mL), moderate (EC_50_ between 100 ng/mL and 1 μg/mL), low (EC_50_ between 1 μg/mL and 100 μg/mL) and N.B. (EC_50_ >100 μg/mL). **(B)** Correlation between binding activity to the SARS-CoV S protein and duration of the immune response. Correlation analysis was performed using the Spearman test, and the r and P values of the correlation are indicated. **(C)** Distribution of SARS-CoV binding mAbs in the VH region (germline). **(D, E)** Comparison of VH and JH mutations between SARS-CoV S protein-binding mAbs and non-SARS-CoV S protein-binding mAbs. The black line denotes the mean value. **(F, G)** Neutralizing capacities against a SARS-CoV pseudovirus and SARS-CoV-2 pseudovirus are shown in the context of binding activity. Color varies with the neutralizing capability, with deep blue and deep red indicating a strong neutralizing capability.

Due to the 76% sequence identity between SARS-CoV S protein and SARS-CoV-2 S protein, there may be conserved epitopes. Accordingly, humoral immunity could utilize these conserved epitopes to produce cross-reactive mAbs response. In this study, 50.6% of SARS-CoV-2-specific antibodies also bound to SARS-CoV S protein, of which 11 (14.3%) recognized SARS-CoV S protein with strong binding activity (EC_50_< 1 μg/mL) (**Figure 2A** and **Figure S5B**). As expected, these cross-binding mAbs also showed continuous affinity maturation (**Figure 2B**). In terms of genetic characteristics, it was observed that mAbs encoded by some VH germline genes, such as VH1-69, VH3-13, VH3-30 and VH4-46, had a tendency to broadly react with SARS-CoV and SARS-CoV-2 (**Figure 2C**). Additionally, cross-binding mAbs showed a tendency for lower SHM in the VH and JH regions, which might limit their affinity maturation, putatively due to the lower exposure of corresponding conserved antigenic sites (**Figures 2D, E**). Overall, these results confirmed an efficient cross-binding antibody response during SARS-CoV-2 infection and the presence of conserved antigenic sites inducing the maturation of cross-reactive mAbs.

Notably, the majority of mAbs with potent neutralizing activity against SARS-CoV-2 showed no reactivity with SARS-CoV, confirming that the unique antigenic sites in SARS-CoV-2 RBD, as immunodominant sites, more efficiently elicit high-affinity mAbs with potent neutralizing capacity than the conserved sites (**Figure 2F**). Subsequently, cross-binding mAbs were tested for their ability to neutralize SARS-CoV. Our results indicated that only P10-6G3, P07-4D10, P05-6H7 and P05-5B6 were identified as cross-neutralizing mAbs against both SARS-CoV and SARS-CoV-2, and P10-6G3 displayed higher binding activity to the SARS-CoV-2 S protein than the SARS-CoV S protein (**Figures 2F, G**). Combined with the weak binding activity, low affinity for SARS-CoV S protein is likely to be the reason why a large number of cross-binding antibodies could not neutralize SARS-CoV (**Figure 2G**). As the antigenic sites recognized by these cross-neutralizing mAbs were common between SARS-CoV and SARS-CoV-2, they could become the key targets for design of universal vaccines against SARS-like coronaviruses and selection of broad therapeutic antibodies.

### Functional characterization of nAbs recognizing multiple antigenic sites

**Figure 3:**
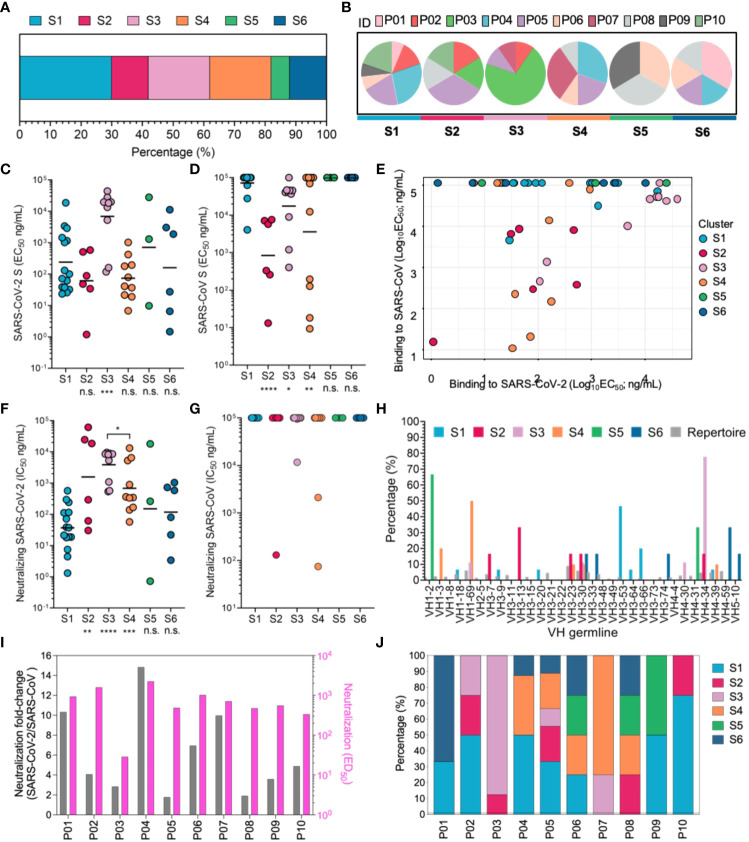
Mapping of multiple neutralizing epitopes recognized by mAbs by cluster analysis and functional characterization. **(A)** Based on cluster analysis of competitive ELISA data for nAbs, nAbs were classified into six clusters targeting six different RBD antigenic sites (S1-6). The percentages of nAbs recognizing different antigenic sites were calculated and are displayed. Colors indicate different individuals. **(B)** Individual composition analysis of nAbs targeting different antigenic sites. ID denotes different convalescent individuals marked by colors. **(C, D)** The binding activity to the SARS-CoV and SARS-CoV-2 S proteins were determined by ELISA and are denoted as EC_50_ values. **(E)** Analysis of antigenic sites recognized by cross-binding nAbs. **(F, G)** Neutralizing capacity against a SARS-CoV-2 pseudovirus **(F)** and SARS-CoV pseudovirus **(G)**, with comparison of nAbs targeting S2-6 and S1-directed nAbs. **(H)** The VH germline genes of each cluster of neutralizing antibodies were analyzed. Different colors are used to indicate each neutralizing cluster. **(I)** Fold increase in ED_50_ of plasma against SARS-CoV-2 pseudoviruses, relative to SARS-CoV-2 pseudoviruses, presented as a pink histogram, and gray histogram indicates neutralization against SARS-CoV-2 pseudoviruses. **(J)** Composition of antibodies targeting different antigenic sites in convalescent COVID-19 patients. Different colors indicated antigenic sites targeted by mAbs of convalescent COVID-19 patients. For panels **(C, D, F, G)**, data are plotted as the geometric mean. n.s., no significant difference; *P < 0.05; **P< 0.01; ***P < 0.001. The black line indicates the mean value. Statistical significance in **(C, D, F, G)** was determined using the Mann-Whitney U test. ****P < 0.0001.

In order to further explore the function of each antigenic sites in RBD, nAbs were classified into six clusters (C1-6) using competition binding assay, and the antigenic sites for C1-6 nAbs were termed as sites S1-6, respectively (**Figures S10**, **S11** and **Figure 3A**). Site S1 should be immunodominant antigenic sites, because the C1 nAbs accounted for relatively higher proportion (**Figure 3A**). Notably, nAbs in different clusters were efficiently induced for majority of convalescent SARS-CoV-2 patients, which embodied common immunogenic characteristics of these antigenic sites in RBD and indicated that SARS-CoV-2 infection can elicit antibody response using similar model (**Figure 3B**).

To determine the functional characteristics of nAbs targeting different sites, we analyzed the biochemical properties of these nAbs. The C1, C2, C4, C5 and C6 nAbs bound to SARS-CoV-2 S protein with comparable EC_50_ values; however, the nAbs elicited by site S3 displayed lower binding activity to SARS-CoV-2 S protein (**Figure 3C**). Furthermore, the majority of C2, C3 and C4 nAbs showed cross-reactivity with SARS-CoV S protein and SARS-CoV-2 S protein, suggesting that these sites were conserved between SARS-CoV and SARS-CoV-2 (**Figures 3D, E**). Nevertheless, these conserved sites in the context of natural infection of SARS-CoV-2 just induced four potent cross-neutralizing mAbs (P10-6G3 targeting site S2, P07-4D10 targeting site S3, and P05-5B6 and P05-6H7 targeting site S4) against SARS-CoV and SARS-CoV-2, since most cross-binding nAbs showed obviously weaker affinity for SARS-CoV S protein than SARS-CoV-2 S protein, leading to inability to achieve cross-neutralization (**Figures 3C–G**). Notably, the conserved sites-directed nAbs showed lower neutralization capacity against SARS-CoV-2 than nAbs recognizing SARS-CoV-2 unique antigenic sites (sites S1, S5 and S6), except those targeting conserved site S3; based on the premise that all nAbs possess similar binding activity to the SARS-CoV-2 S protein, these nAbs targeting conserved sites are implied to have a disadvantage in blocking S protein binding to receptor ACE2, putatively due to less overlap between their antigenic sites and ACE2 footprint (**Figures 3C, F**). Markedly, C1 nAbs isolated from the overwhelming majority of convalescent individuals recognized antigenic site S1, and potently and specifically inhibited SARS-CoV-2 infection by blocking S protein attachment to ACE2, revealing that site S1 is an immunodominant antigenic site in SARS-CoV-2 RBD that efficiently elicits a strong NAb response during natural SARS-CoV-2 infection (**Figures 3B, F** and **Figure S12A**). The immunodominance of site S1 may result from either its accessibility in different conformations of SARS-CoV-2 S protein or the innate affinity of the corresponding C1 nAbs derived from the naïve B cell repertoire (VH 3-53/66 germline) (**Figure 3H**); the latter factor would lead to rapid affinity maturation of C1 mAbs without the need for a high level of SHM (31, 43, 44). In contrast, while large amplification of the same antibody clone against site S3 was exhibited in the convalescent individual P03, the nAbs derived from this antibody clone poorly inhibited SARS-CoV-2 infection, revealing that site S3 is possibly an immunodominant, but weakly neutralizing site (**Figure 3F**, **Figure S6**, **Figure 1A** and **Figure S4**). The characteristics of nAbs targeting different antigenic sites is further mapped to plasma neutralizing function. nAbs recognizing conserved antigenic site S3 showed significant clonal amplification in P03, which resulted in weaker plasma neutralization compared to the other COVID-19 patients. High proportion of specific antibodies against site S1 can ensure stronger neutralization activity against SARS-CoV-2 in COVID-19 patient plasma, but its cross-neutralization potency decreased significantly, and P04 was the most representative among these convalescent patients (**Figures 3I, J**). On the contrary, P05 and P08 plasma showed higher cross-neutralization potency, which might result from the numerous cross-neutralizing mAbs binding to the conserved antigenic sites. Thus, our findings confirm that conserved antigenic sites can broadly induce antibody response in COVID-19 patients, while cross-neutralization potency varies for different patients.

Taken together, sites S2, S3 and S4 were identified as conserved antigenic sites between SARS-CoV-2 and SARS-CoV that could induce cross-neutralizing antibody response and should be considered in the rational design of universal SARS-like coronavirus vaccines, and the remaining sites were unique antigenic sites for SARS-CoV-2. The difference in binding location possibly confer nAbs elicited by conserved antigenic sites might show weaker neutralization of SARS-CoV-2 than those elicited by unique antigenic sites.

### Identification of antigenic sites S1-6 in RBD

**Figure 4:**
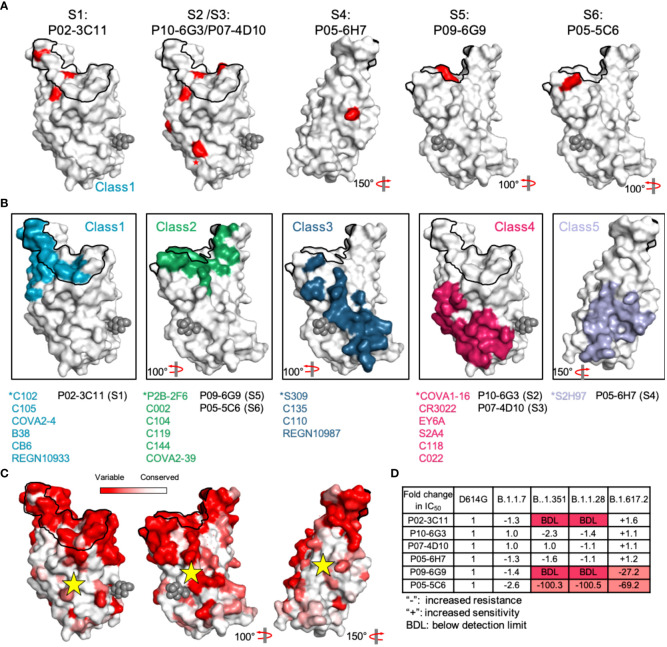
Dynamic analysis of site S1-6 accessibility. **(A)** Relative positions of sites S1-6 in the RBD. Red indicates the critical residues interfering the binding activity of representative nAbs. **(B)** Some SARS-CoV-2 RBD-specific mAbs reported in other studies are displayed in different colors, and cyan, green, blue, claret and purple indicate the mAbs C102, P2B-2F6, S309, COVA1-16 and S2H97, respectively. These mAbs were identified as representative mAbs for Classes 1-5. ACE2 footprint is outlined in black line. The glycan at position N343 is rendered as black spheres. **(C)** Conservation analysis of antigenic sites-directed by candidate mAbs using sequences including SARS-CoV-2 (n = 2216,010) and other Sarbecoviruses (n = 80). Deeper white indicates the more conserved region that was marked by a yellow star. **(D)** The fold change of mAbs neutralization potency against SARS-CoV-2 variants (B.1.1.7, B.1.351, B.1.1.28 and B.1.617.2), compared to that against the D614G variant.

It is more helpful to understand the functional characteristics of corresponding antibodies by analyzing the structural characteristics of each antigenic site. To determine the spatial position of sites S1-6, we performed a mutagenesis study by substituting ACE2-interactive and noninteractive residues with alanine or arginine in RBD, and then assessed the decreased binding activity of representative nAbs to mutant RBDs compared to the reference RBD (45). The reduction in binding activity to each mutant RBD is shown in **Figure S14**. The spatial positions of all antigenic sites in RBD were simultaneously displayed to demonstrate their relative locations, which is critical to elucidate the functional characteristics of all neutralizing antigenic sites in RBD (**Figure S15A** and **Figure 4A**). Fortunately, numerous key epitopes in RBD have been identified by antigen-antibody complex structure and could be classified into five classes, which provides an important reference for this study (4, 28, 46). Site S1, S2 (and S3), S4 and S5 (and S6) sites are similar to Class 1, Class 4, Class 5 and Class 2/3 sites, respectively, according to their interactive residues and neutralizing characteristics of corresponding mAbs (**Figures 4A, B**).

SARS-CoV-2 unique sites S1, S5 and S6 largely overlapped with ACE2 footprint, supporting potent neutralizing activities of corresponding nAbs by efficiently blocking S protein binding to receptor, whereas conserved sites S2, S3 and S4 distant from ACE2 footprint is not conducive for neutralization (**Figure S15A**). However, site S4-specific nAbs, similar to broadly neutralizing mAb S2H97, might neutralize SARS-CoV-2 and SARS-CoV by promoting direct shed of S1 subunit, rather than blocking RBD attachment to ACE2. Site S2, S3 and S4 are highly conserved among SARS-CoV, SARS-CoV-2 and even other SARS-related viruses (**Figure 4C** and **Figure S15B**). Thus, nAbs targeting these antigenic sites retain strong neutralization potency against SARS-CoV-2 variants (B.1.1.7, B.1.351, B.1.1.28 and B.1.617.2), nevertheless, these variants escape neutralization of representative nAbs targeting SARS-CoV-2 unique sites (**Figure 4D**). As some neutralizing antigenic sites are hidden by the RBD in the lying-down state, this masking becomes an important immune escape mechanism of SARS-CoV-2 (41). The conserved sites, especially site S3, were highly concealed in the RBD lying-down state by adjacent RBD monomers. Even when the RBD was in the standing-up state, site S3 was not sufficiently exposed, and this inadequate exposure failed to improve the affinity maturation of S3-directed mAbs (**Figure S16**). Similarly, insufficient space for nAbs binding to conserved site S4 was found on the closed S protein, and only the RBD in the standing-up state could improve the accessibility of site S4.

Thus, our findings indicate that conserved antigenic sites display less accessibility than SARS-CoV-2 unique epitopes and that poor accessibility hinders the affinity maturation of site S3-directed mAbs in natural infection and even might decrease the cross-neutralizing antibody response after SARS-CoV-2 vaccination.

### Rational design of SARS-CoV-2 RBD to enhance the immunodominance of conserved antigenic sites

**Figure 5:**
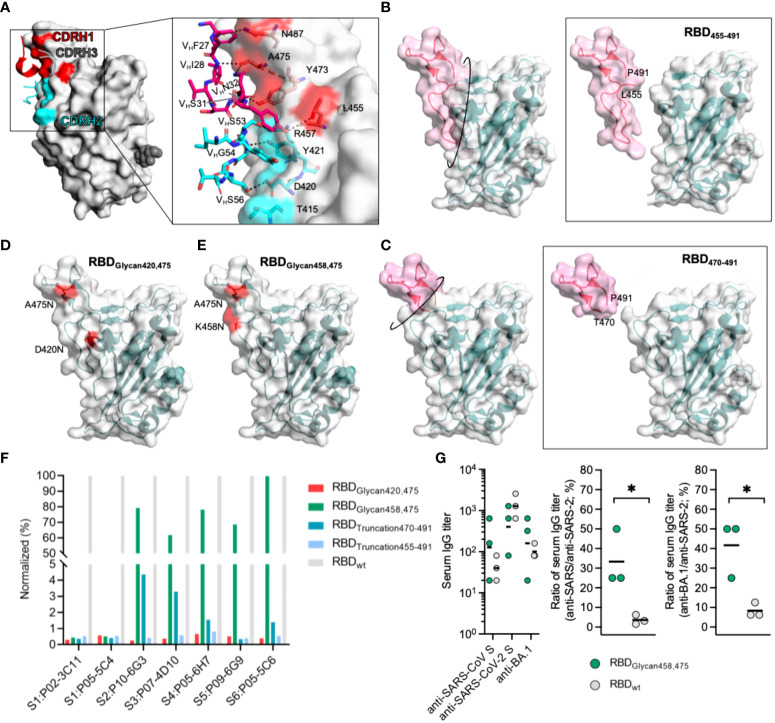
Design of SARS-CoV-2 RBD variants to improve the competitiveness of subdominant conserved antigenic sites. **(A)** Interaction between CDRH1 and CDRH2 residues and the SARS-CoV-2 RBD. Residues of mAbs constructing hydrogen bonds with CDRH1 and CDRH2 in the RBD are rendered as red and cyan sticks. **(B–E)** Design of a SARS-COV-2 RBD with an enhanced capability to elicit cross-reactive antibodies. A truncated SARS-CoV-2 RBD was produced by removing T455-P491 and termed RBD_Truncation455-491_ in **(B)**, and the RBD produced by removing T470-P491 was termed RBD_Truncation470-491_ in **(C)**. A SARS-CoV-2 RBD glycosylated at positions 420 and 475 was termed RBD_Glycan420,475_ in **(D)**, and an RBD glycosylated at positions 458 and 475 was termed RBD_Glycan458,475_ in **(E)**. **(F)** Influence of designed RBDs on the binding activity of representative nAbs targeting different antigenic sites. **(G)** Antibody response against the SARS-CoV S protein and SARS-CoV-2 S protein induced by RBD_Glycan458,475_ in mice (N=3). BALB/c mice were immunized with 20 μg/dose at week 0, and specific IgG titers in the serum were tested at week 2. The ratio of the serum IgG titer against the SARS-CoV S protein to that against the SARS-CoV-2 S protein was calculated. For panel **(G)**, data are plotted as the geometric mean or mean. Statistical significance in **(G)** was determined using a nonpaired t test, and * indicates P < 0.05.

The majority of mAbs recognizing antigenic site S1 were VH3-53/66 mAbs with a short (mostly 11 residues) CDRH3 sequence. Moreover, many studies have also reported the same enrichment of VH3-53/66 mAbs targeting antigenic sites similar to site S1 and found that they share structural similarities with each other (8, 29, 46–48). These VH3-53/66 mAbs showed native binding to SARS-CoV-2 RBD residues using CDRH1 and CDRH2 by forming many hydrogen bonds (L455, Y473, A475 and N487 bound by CDRH1, and D420, Y421 and R457 bound by CDRH2) (**Figure 5A** and **Figure S17**). Therefore, the native binding advantage between antigenic site S1 and VH3-53/66 was the cardinal cause of site S1 immunodominance, which could result in massive amplification of antigenic site S1-specific B cells and competition to inhibit the proliferation of B cells directed against conserved antigenic sites. To indirectly enhance the competitiveness of conserved antigenic sites in the immune response, it is essential to decrease the immunodominance of site S1. To this end, we designed a variety of SARS-CoV-2 RBD variants with antigenic site S1 silencing using either protein truncation or glycan modification (**Figures 5B–E**). Glycan modification at positions K458 and A475 within site S1, termed RBD_Glycan458,475_, was successful in destroying the binding of S1-directed mAbs, including P02-3C11 derived from VH3-66 and P05-5C4 derived from VH3-53, and could maintain conserved antigenic sites S2, S3 and S4 (**Figure 5F**). To preliminarily investigate whether RBD_Glycan458,475_ could improve the cross-binding antibody response, BALB/c mice were immunized with 20 μg/dose RBD_Glycan458,475_. Two weeks after immunization, the mice receiving RBD_Glycan458,475_ or the reference RBD all presented detectable serum anti-SARS-CoV S IgG and anti-SARS-CoV-2 S IgG; however, RBD_Glycan458,475_ induced a significantly higher cross-binding IgG titer than the reference RBD (**Figure 5G**). Additionally, the results also revealed that RBD_Glycan458,475_ could induce higher cross-binding IgG titer against SARS-CoV-2 and the Omicron variant (BA.1) than the reference RBD (**Figure 5G**). Hence, universal vaccines based on such glycan modification of the SARS-CoV-2 RBD have the potential to induce a stronger cross-binding antibody response that could efficiently protect against infection by SARS-like coronaviruses and emerging SARS-CoV-2 variants.

## Discussion

RBD is defined as the immunodominant domain within the SARS-CoV-2 S protein (4), which was supported by its few glycosylation sites compared with the other S protein domains and higher accessibility within the S protein with variable conformations, as well as by S1 domain shedding (6, 28). Structural studies have proven that the S protein possesses conformational dynamics, in which different prefusion conformations expose a variety of crucial antigenic sites, including conserved antigenic sites between SARS-CoV and SARS-CoV-2 (31, 49). Although some conserved antigenic sites identified by cross-binding mAbs have been reported, a systematic analysis is still lacking, which is prejudicial to rational design of universal vaccines (4–8). We used information obtained from neutralizing mAbs isolated from convalescent SARS-CoV-2 patients to develop a quantitative antigenic map of SARS-CoV-2 RBD neutralizing sites that demonstrates immunodominance, neutralization properties and conserved properties. Six dominant antigenic sites were identified, of which sites, S2, S3 and S4 are conserved antigenic sites and can elicit cross-neutralizing antibody response to SARS-CoV and SARS-CoV-2. Analysis of difference in plasma neutralization capacity from the perspective of mAbs is more conducive to the cognition of the process of humoral immune response to SARS-CoV-2, so as to promote the directional induction of functional immune response. This study demonstrates that, although similar plasma neutralization against SARS-CoV-2 was determined among convalescent COVID-19 patients, there was significant difference in cross-neutralization activity against SARS-CoV, which was probably caused by the diverse strength of antibody response to different antigenic sites. High proportion of conserved antigenic sites-specific antibodies will significantly decrease plasma neutralization titer, but accompanied by strong cross-neutralization capacity, which gives us the hope of developing universal vaccine based on the conserved antigenic sites, but also presents a serious test of how to effectively enhance the corresponding immune response.

These conserved antigenic sites are subdominant, and S3 induces a lower-affinity mAb response than unique antigenic site S1 that is immunodominant and coincides with the ACE2 footprint; this hierarchy is putatively related to the lower accessibility of conserved antigenic sites in a variety of conformations. Nevertheless, conserved antigenic sites can still effectively induce affinity maturation of specific antibodies, which provides an important basis for vaccine design based on conserved antigenic sites. SARS-CoV-2 unique antigenic sites (S1, S5 and S6) efficiently induce a specific antibody response and inhibit the production of cross-binding antibodies. Additionally, antigenic site S1 with native binding advantage of antibodies derived from VH3-53/66 might further suppress the humoral immune response to conserved antigenic sites through the depletion of a large number of B cells. Therefore, these antigenic sites, especially site S1, should be silenced for universal vaccine design. Moreover, some predominant SARS-CoV-2 variants, including B.1.351 and B.1.1.28 with K417N, E484K and N501Y mutations causing changes in antigenic sites overlapping with unique antigenic sites S1, S5 and S6, promote evasion of antibody-mediated immunity obtained by natural infection or vaccination; however, no cross-binding mAbs displaying decreased binding activity to these variants have been reported (20, 22–24). These findings prove that it is difficult for universal vaccines based on unique antigenic sites in the RBD to induce conserved antibody responses to prevent the possible pandemic risks of persistent SARS-CoV-2 variants with antigenic drift or SARS-like coronaviruses in the future; nevertheless, focusing on conserved antigenic sites might have great potential for universal SARS-like coronavirus vaccines (50).

Similar strategies for universal vaccine design have been proposed for the development of universal influenza virus vaccines that protect against infection with seasonal drift and novel pandemic influenza virus strains (51, 52). Candidates for universal influenza vaccines are mainly based on the conserved antigenic sites in the stalk domain of hemagglutinin, for example, headless hemagglutinin structures and display of conserved stalk epitopes on nanoparticles, which has shown promising results in animal models and has great reference significance for SARS-CoV-2 vaccines (53–56). To indirectly enhance the competitiveness of conserved sites in the mAb response by decreasing the immunodominance of site S1, we designed a variety of SARS-CoV-2 RBD proteins with site S1 silencing by either removal of a peptide fragment or glycan modification. In this study, the designed protein RBD_Glycan458,475_ with a glycan modification destroying site S1 and maintaining the remaining conserved sites induced stronger cross-binding antibody response, revealing that such a SARS-CoV-2 RBD design could promote the development of universal vaccines against SARS-like coronaviruses. To further enhance the immunogenicity, the modified RBD could be display on particle, such as ferritin (57), mi3 (58) and I53 (59).

In summary, our studies defined a quantitative antigenic map of neutralizing sites within SARS-CoV-2 RBD and completed the characterization of conserved antigenic sites, which is required for rational design of universal vaccines. Moreover, we tried to design RBD proteins to enhance the immune competitiveness of conserved antigenic sites. Although SARS-CoV-2 vaccines have been developed and approved, our long-term efforts aimed at preparing universal vaccines for other human epidemics caused by SARS-CoV-2 VOCS and SARS-like coronaviruses that may become prevalent in the future are still necessary.

## Data availability statement

The original contributions presented in the study are included in the article/**Supplementary Material**. Further inquiries can be directed to the corresponding authors.

## Ethics statement

The studies involving human participants were reviewed and approved by School of Public Health. The patients/participants provided their written informed consent to participate in this study.

## Author contributions

SW, QY, HY, TZ, ZZ, and NX contributed to the experimental design. SW, DW, QY, HX, JW, TZ, ZZ, and NX participated in discussion and interpretation of the results. SW, DW, HX, JW, TZ, ZZ, and NX contributed to the manuscript preparation. SW, HX, JW, ZT, ZC, YZ, DY, XL, CL, SG, YL, and XZ contributed to the preparation and *in vitro* characterization of antibody. YW and WT performed the animal experiments. All authors contributed to the article and approved the submitted version.

## Funding

This work was supported by the National Natural Science Foundation of China (81993149041 for NX; 81871316 for QY). The Science and Technology Major Project of Fujian Province (grant number 2020YZ014001). Xiamen Science and Technology Major Project (grant number 3502Z2020YJ02).

## Conflict of interest

The authors declare that the research was conducted in the absence of any commercial or financial relationships that could be construed as a potential conflict of interest.

## Publisher’s note

All claims expressed in this article are solely those of the authors and do not necessarily represent those of their affiliated organizations, or those of the publisher, the editors and the reviewers. Any product that may be evaluated in this article, or claim that may be made by its manufacturer, is not guaranteed or endorsed by the publisher.

## Supplementary material

Supplementary Figure 1 Characteristics analysis of humoral immune response by COVID-19 convalescent plasma. **(A)** Correlation between days after symptom onset and plasma antibody titer including anti-RBD antibody, anti-RBD IgG and anti-RBD IgM, using Spearman correlation test. **(B)** Correlation test between anti-RBD titers and PSV SARS-CoV-2 neutralizing capacity are determined by Spearman correlation test. r and P values of the correlation are indicated.

Supplementary Figure 2 Identification of SARS-CoV-2 RBD-specific memory B cells and isolation of SARS-CoV-2 RBD-specific antibodies. **(A)** SARS-CoV-2 RBD-specific memory B cells are identified as CD3-/CD19+/CD27+/SARS-CoV-2 RBD+, and the percentage of RBD-specific B cells is indicated. **(B, C)** The BCR (B cell receptor) subtypes of RBD-specific memory B cells are analyzed by goat anti-human IgG and goat anti-human IgM, then are statistically analyzed. **(D)** Recombinant monoclonal antibodies with SARS-CoV-2 RBD specificity are identified by ELISA. Gray line indicates limitation of anti-RBD antibodies detection.

Supplementary Figure 3 Phylogenetic analysis of heavy chain gene of SARS-CoV-2 RBD-specific mAbs. Maximum-likelihood phylogenetic tree of fully heavy chain of RBD-specific antibodies (N=77). Each color represents heavy chain sequence of SARS-CoV-2 RBD-specific mAbs from different convalescent individuals.

Supplementary Figure 4 Gene repertoire analysis of SARS-CoV-2 RBD-specific mAbs. V gene frequencies for heavy chain **(A)** and light chain **(B)** of SARS-CoV-2 RBD-specific antibodies. Colors indicate different convalescent individuals. Germline of VH is determined using the Immunogenetics (IMGT).

Supplementary Figure 5 Binding activity to S protein and neutralizing capacity against SARS-CoV-2 pseudovirus of SARS-CoV-2 RBD-specific mAbs. **(A, B)** Binding activity of mAbs to SARS-CoV S protein are compared among individual in B, and to SARS-CoV-2 S protein in C. Black line indicates mean value of EC_50_. **(C, D)** Neutralizing capacity of mAbs against SARS-CoV-2 are compared among individual in **(C)** and blocking capacity of mAbs in **(D)**. Neutralizing capacity are tested by SARS-CoV-2 pseudovirus. Blocking assay is performed by incubating mixture of antibodies and SARS-CoV-2 S protein with ACE2-expressing cells. Black line indicates mean IC_50_.

Supplementary Figure 6 Neutralization of SARS-CoV-2 RBD-specific mAbs against SARS-CoV-2 pseudovirus. Red indicates mAbs obtained from P03 convalescent individual.

Supplementary Figure 7 The correlation between neutralization potency and blocking capability of SARS-CoV-2 RBD-specific mAbs. The scatter plot depicting neutralizing capacity and blocking capacity of specific mAbs from different individuals annotated by colors.

Supplementary Figure 8 Analysis of CDRH3 length of antibodies derived from VH 3-53/66. **(A)** Repertoire information of RBD-specific antibodies composed of VH 3-53/66. **(B)** Length distribution of CDRH3 for RBD-specific antibodies derived from VH 3-53/66 by comparison with the remaining VH germline encoding antibodies. **(C)** Correlation of CDRH3 length and binding activity to SARS-CoV-2 S protein is performed for specific antibodies derived from of VH 3-53/66.

Supplementary Figure 9 Correlation analysis for days after symptom onset and mean binding activity of SARS-CoV-2 RBD-specific mAbs from corresponding convalescent individuals using Spearman correlation test. r and P values of the correlation are indicated.

Supplementary Figure 10 Competition ELISA for neutralizing mAbs. Competition ELISA is performed by using naked mAbs to block HRP-coupled mAbs, and ELISA signal for each HRP-coupled mAb is normalized to the signal in the absence of naked mAbs. The heat map of competition ELISA data is shown, with parameters colored continuously from white (0, corresponding to 0% inhibition) to red (4, corresponding to 93.7% inhibition) in the scale bar.

Supplementary Figure 11 Epitope mapping of mAbs by clustering analysis and functional characterization. By competition ELISA data, neutralizing mAbs are clustered into six group, Cluster1-6, and corresponding epitopes to each mAb cluster are defined as Site1-6. The color ranging from red to blue represented blocking potency against other antibodies (4.321 corresponding to 95% blocking rate and 0.074 corresponding to 5% blocking rate). The source of information and neutralization potency of each mAb are also indicated by different colors.

Supplementary Figure 12 Analysis of blocking capability against SARS-CoV-2 S protein binding to ACE2. **(A)** Blocking capacity of nAbs targeting sites S2-6 are compared with that of nAbs recognizing S1. **(B)** The neutralizing capacity and blocking capacity of nAbs recognizing site S4 are analyzed, and nAbs ID are indicated in figure.

Supplementary Figure 13 Neutralization capacity of a combination of representative nAbs targeting sites S1-6 against the SARS-CoV-2 pseudovirus and SARS-CoV-2 pseudovirus.

Supplementary Figure 14 Identification of SARS-CoV-2 RBD critical residues recognized by nAbs using selected amino acid substitution. **(A)** Mutate residues of SARS-CoV-2 RBD shown in pink. **(B)** Mutation of residues leading to damaging effect on SARS-CoV-2 RBD activity. Dash line indicates 25% binding activity of mutant RBD relative to wild type RBD. **(C)** The selected amino acid of RBD is mutated to alanine or arginine on purpose. Binding activity of sites S1-6 representative mAbs to wild-type (WT) and mutant RBD was measured by ELISA. The binding capacity to mutate RBD is normalized by binding to wild type RBD. Lines denote 10% binding activity relative to wild type RBD and 25% binding activity relative to wild type RBD. Residues reducing binding activity by more than 75% are identified critical residues for representative nAbs.

Supplementary Figure 15 Identification of sites S1-6 spatial position. **(A)** Structure of the RBD highlighting the critical residues interfering binding activity of representative nAbs, red denotes residues reducing binding activity by more than 75%. **(B)** Conservative analysis of sites S1-6, carmine denotes different residues between SARS-CoV-2 RBD and SARS-CoV RBD.

Supplementary Figure 16 CDRH1-3 sequence analysis of mAbs derived from IGHV3-53/66, including P05-5C4 derived from IGHV3-53 and P02-3C11 derived from IGHV3-66 targeting site S1.

Supplementary Table 1 Information of COVID-19 convalescent individuals. F, female; M, male.

Supplementary Table 2 Plasma anti-RBD antibody titers and neutralization capacity for COVID-19 convalescent individuals.

Supplementary Table 3 Amino acid sequence of diverse SARS-CoV-2 RBD variants expressed.
