# Supplementary material for: Potential of conserved antigenic sites in development of universal SARS-like coronavirus vaccines
Source: Front Immunol. 2022 Sep 20;13:952650. doi: 10.3389/fimmu.2022.952650 (PMC9530325; doi:10.3389/fimmu.2022.952650)
Supplement: Supplementary Figure 1 — Characteristics analysis of humoral immune response by COVID-19 convalescent plasma. (A) Correlation between days after symptom onset and plasma antibody titer including anti-RBD antibody, anti-RBD IgG and anti-RBD IgM, using Spearman correlation test. (B) Correlation test between anti-RBD titers and PSV SARS-CoV-2 neutralizing capacity are determined by Spearman correlation test. r and P values of the correlation are indicated. [file DataSheet_1.docx]

**Supplemental figure legends**

**Figure S1. Characteristics analysis of humoral immune response by COVID-19 convalescent plasma.** **(A)** Correlation between days after symptom onset and plasma antibody titer including anti-RBD antibody, anti-RBD IgG and anti-RBD IgM, using Spearman correlation test. **(B)** Correlation test between anti-RBD titers and PSV SARS-CoV-2 neutralizing capacity are determined by Spearman correlation test. r and P values of the correlation are indicated.

**Figure S2. Identification of SARS-CoV-2 RBD-specific memory B cells and isolation of SARS-CoV-2 RBD-specific antibodies. (A)** SARS-CoV-2 RBD-specific memory B cells are identified as CD3-/CD19+/CD27+/SARS-CoV-2 RBD+, and the percentage of RBD-specific B cells is indicated. **(B and C)** The BCR (B cell receptor) subtypes of RBD-specific memory B cells are analyzed by goat anti-human IgG and goat anti-human IgM, then are statistically analyzed. **(D)** Recombinant monoclonal antibodies with SARS-CoV-2 RBD specificity are identified by ELISA. Gray line indicates limitation of anti-RBD antibodies detection.

**Figure S3. Phylogenetic analysis of heavy chain gene of SARS-CoV-2 RBD-specific mAbs.** Maximum-likelihood

phylogenetic tree of fully heavy chain of RBD-specific antibodies (N=77). Each color represents heavy chain sequence of SARS-CoV-2 RBD-specific mAbs from different convalescent individuals.

**Figure S4. Gene repertoire analysis of SARS-CoV-2 RBD-specific mAbs.** V gene frequencies for heavy chain **(A)** and light chain **(B)** of SARS-CoV-2 RBD-specific antibodies. Colors indicate different convalescent individuals. Germline of VH are determined using the Immunogenetics (IMGT).

**Figure S5.** **Binding activity to S protein and neutralizing capacity against SARS-CoV-2 pseudovirus of SARS-CoV-2 RBD-specific mAbs. (A and B)** Binding activity of mAbs to SARS-CoV S protein are compared among individual in B, and to SARS-CoV-2 S protein in C. Black line indicates mean value of EC_50_. **(C and D)** Neutralizing capacity of mAbs against SARS-CoV-2 are compared among individual in **C**, and blocking capacity of mAbs in **D**. Neutralizing capacity are tested by SARS-CoV-2 pseudovirus. Blocking assay is performed by incubating mixture of antibodies and SARS-CoV-2 S protein with ACE2-expressing cells. Black line indicates mean IC_50_.

**Figure S6. Neutralization of SARS-CoV-2 RBD-specific mAbs against SARS-CoV-2 pseudovirus.** Red indicates mAbs obtained from P03 convalescent individual.

**Figure S7. The correlation between neutralization potency and blocking capability of SARS-CoV-2 RBD-specific mAbs.** The scatter plot depicting neutralizing capacity and blocking capacity of specific mAbs from different individuals annotated by colors.

**Figure S8.** **Analysis of CDRH3 length of antibodies derived from VH 3-53/66. (A)** Repertoire information of RBD-specific antibodies composed of VH 3-53/66. **(B)** Length distribution of CDRH3 for RBD-specific antibodies derived from VH 3-53/66 by comparison with the remaining VH germline encoding antibodies. **(C)** Correlation of CDRH3 length and binding activity to SARS-CoV-2 S protein is performed for specific antibodies derived from of VH 3-53/66.

**Figure S9.** Correlation analysis for days after symptom onset and mean binding activity of SARS-CoV-2 RBD-specific mAbs from corresponding convalescent individuals using Spearman correlation test. r and P values of the correlation are indicated.

**Figure S10. Competition ELISA for neutralizing mAbs.** Competition ELISA is performed by using naked mAbs to block HRP-coupled mAbs, and ELISA signal for each HRP-coupled mAb is normalized to the signal in the absence of naked mAbs. The heat map of competition ELISA data is shown, with parameters colored continuously from white (0, corresponding to 0% inhibition) to red (4, corresponding to 93.7% inhibition) in the scale bar.

**Figure S11. Epitope mapping of mAbs by clustering analysis and functional characterization.** By competition ELISA data, neutralizing mAbs are clustered into 6 group, Cluster1-6, and corresponding epitopes to each mAb cluster are defined as Site1-6. The color ranging from red to blue represented blocking potency against other antibodies (4.321 corresponding to 95% blocking rate and 0.074 corresponding to 5% blocking rate). The source of information and neutralization potency of each mAb are also indicated by different colors.

**Figure S12. Analysis of blocking capability against SARS-CoV-2 S protein binding to ACE2. (A)** Blocking capacity of nAbs targeting sites S2-6 are compared with that of nAbs recognizing S1. **(B)** The neutralizing capacity and blocking capacity of nAbs recognizing site S4 are analyzed, and nAbs ID are indicated in figure.

**Figure S13. Neutralization capacity of a combination of representative nAbs targeting sites S1-6 against the SARS-CoV-2 pseudovirus and SARS-CoV-2 pseudovirus.**

**Figure S14. Identification of SARS-CoV-2 RBD critical residues recognized by nAbs using selected amino acid substitution. (A)** Mutate residues of SARS-CoV-2 RBD shown in pink. **(B)** Mutation of residues leading to damaging effect on SARS-CoV-2 RBD activity. Dash line indicates 25% binding activity of mutant RBD relative to wild type RBD. **(C)** The selected amino acid of RBD are mutated to alanine or arginine on purpose. Binding activity of sites S1-6 representative mAbs to wild-type (WT) and mutant RBD was measured by ELISA. The binding capacity to mutate RBD is normalized by binding to wild type RBD. Lines denote 10% binding activity relative to wild type RBD and 25% binding activity relative to wild type RBD. Residues reducing binding activity by more than 75% are identified critical residues for representative nAbs.

**Figure S15. Identification of sites S1-6 spatial position.** **(A)** Structure of the RBD highlighting the critical residues interfering binding activity of representative nAbs, red denotes residues reducing binding activity by more than 75%. **(B)** Conservative analysis of sites S1-6, carmine denotes different residues between SARS-CoV-2 RBD and SARS-CoV RBD.

**Figure S16. (A)** Accessibility analysis of site 1, site 5 and site 6 in either the standing-up or lying-down state of the RBD of the S protein. Red, yellow and purple denote critical residues of site S1, site S5 and site S6 in the RBD of the S protein, respectively (PDB codes: 6VYB and 6VXX). **(B)** Accessibility analysis of conserved sites S2, S3 and S4 in either the lying-down state of the RBD of the S protein (PDB code: 6VXX) or the standing-up state of the RBD of the S protein (PDB code: 6VYB). Red, yellow and purple denote critical residues of sites S2, S3 and S4.

**Figure S17.** CDRH1-3 sequence analysis of mAbs derived from IGHV3-53/66, including P05-5C4 derived from IGHV3-53 and P02-3C11 derived from IGHV3-66 targeting site S1.

**Figure S1.
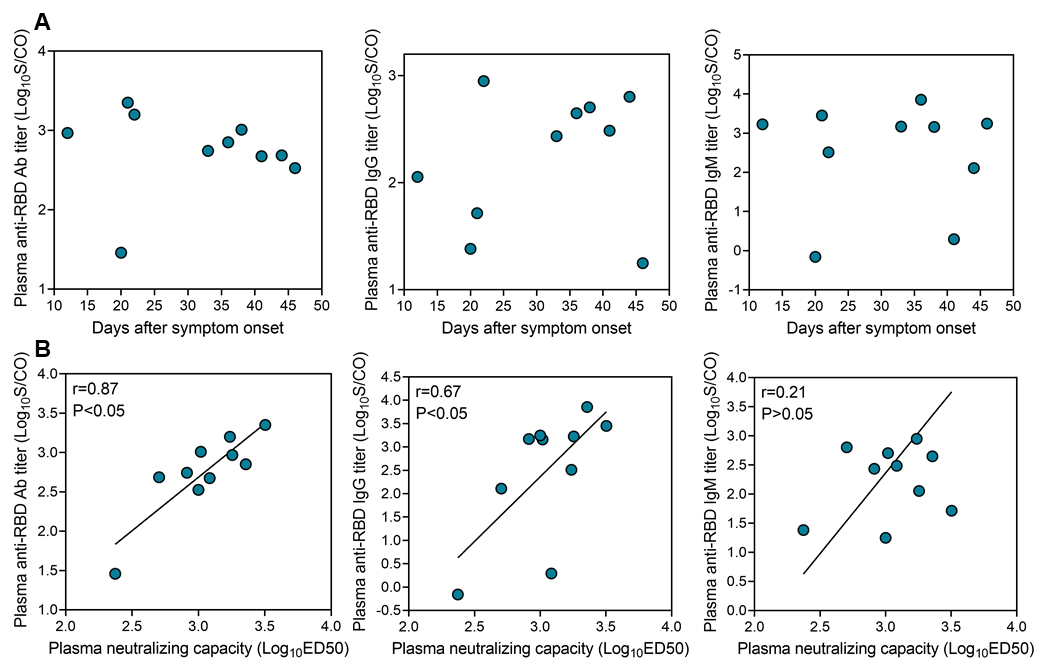
**

**Figure S2.**

**
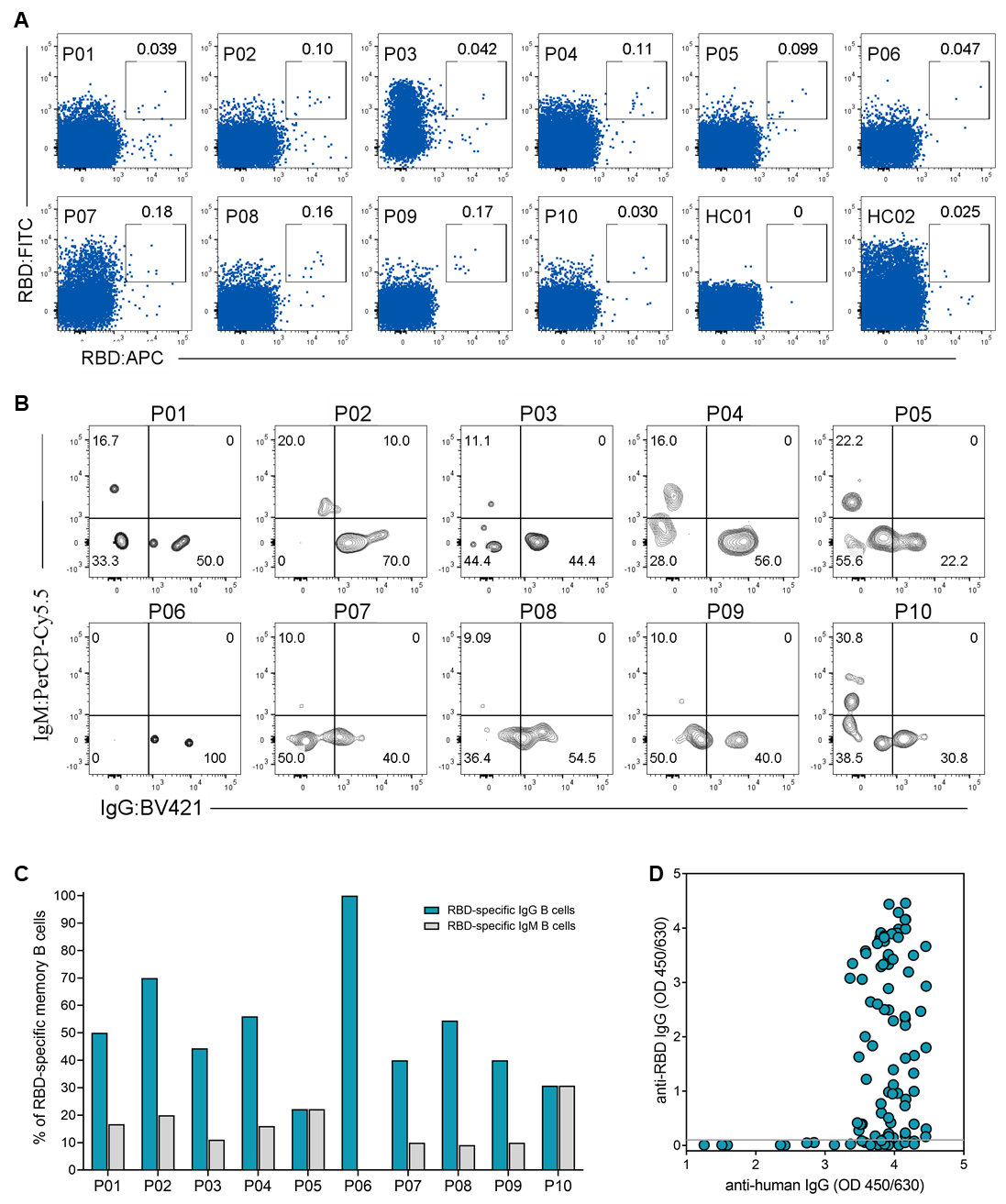
**

**Figure S3.**

**
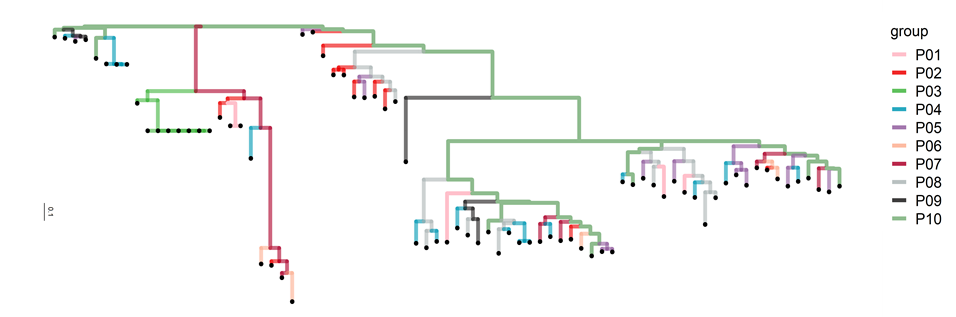
**

**Figure S4.**

**
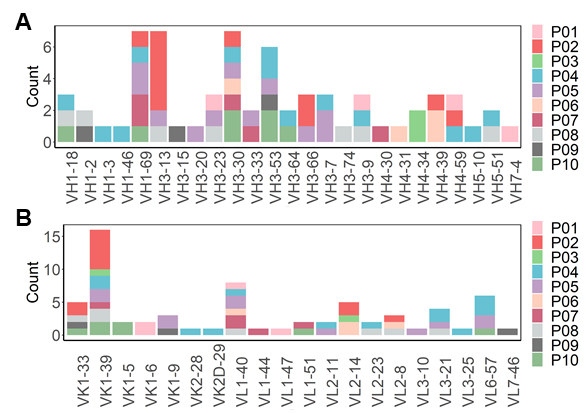
**

**Figure S5.**

**
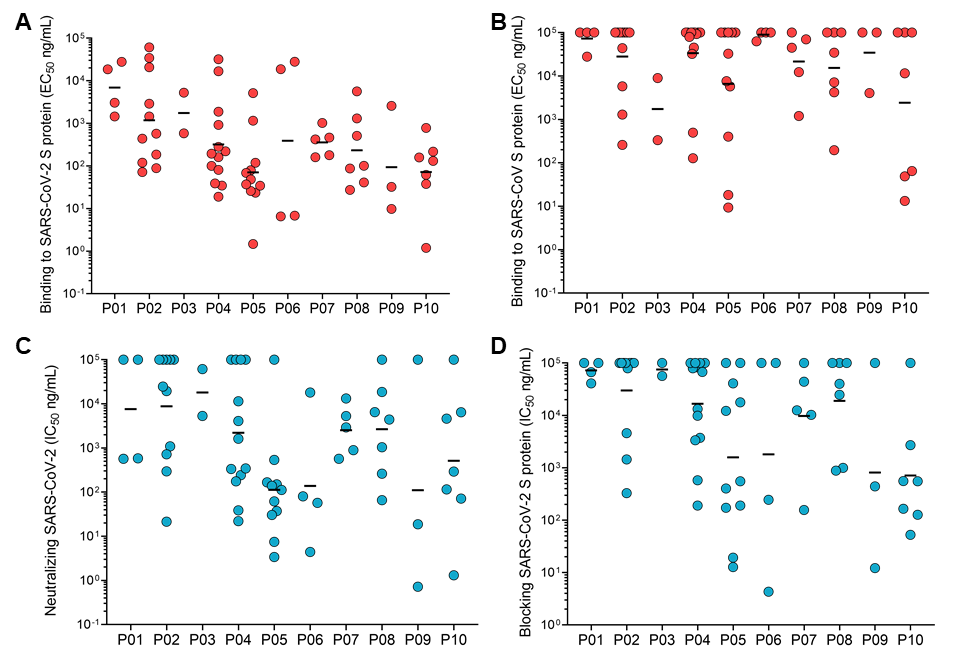
**

**Figure S6.**

**
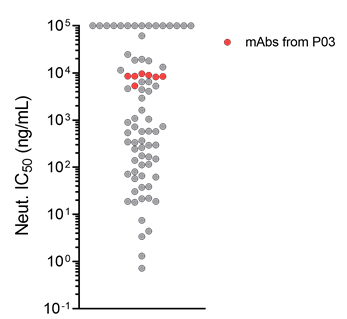
**

**Figure S7.**

**
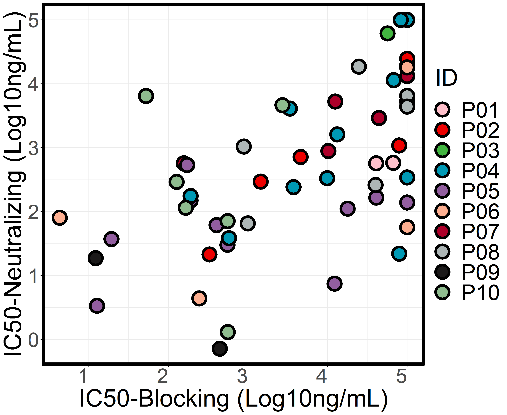
**

**Figure S8.**

**
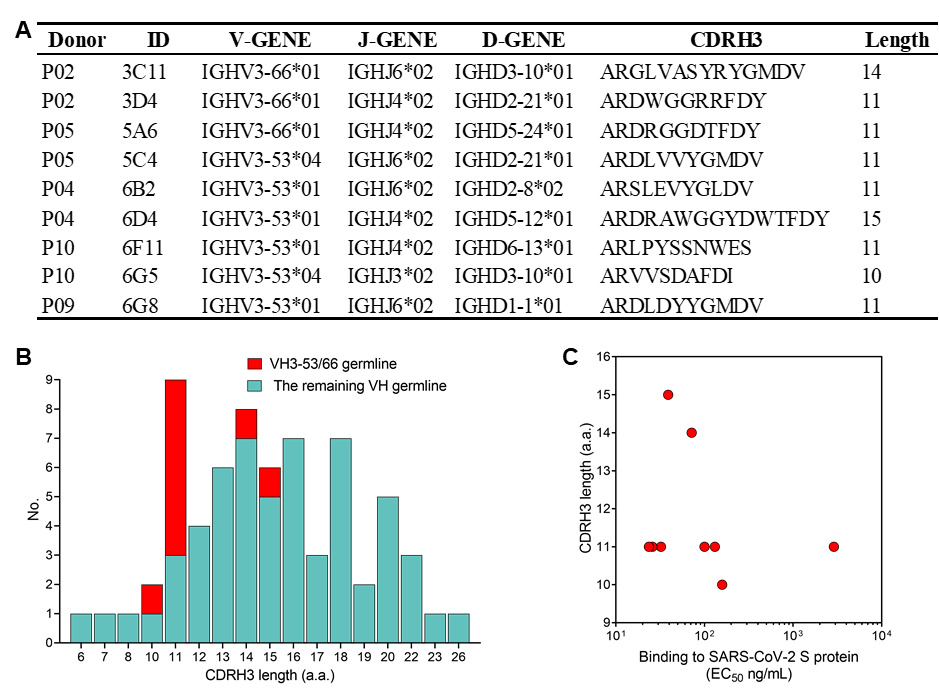
**

**Figure S9.**

**
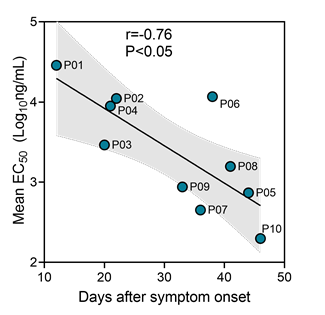
**

**Figure S10.**

**
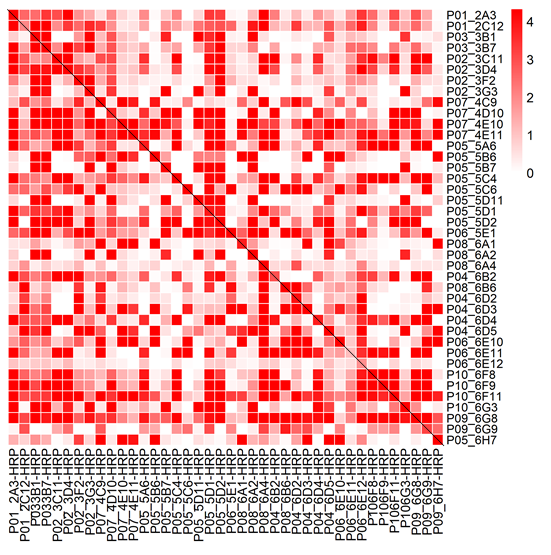
**

**Figure S11.**

**
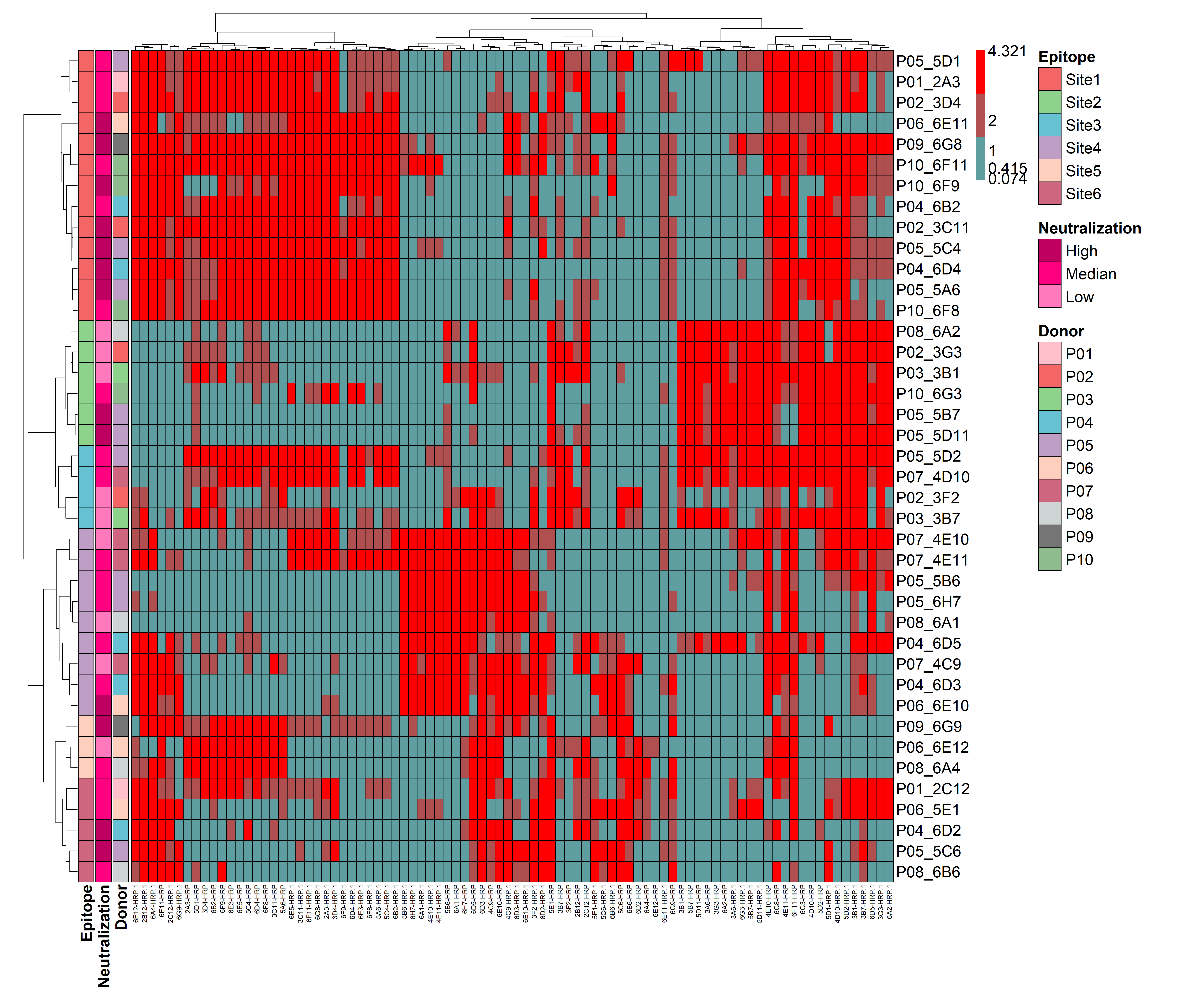
**

**Figure S12.**

**
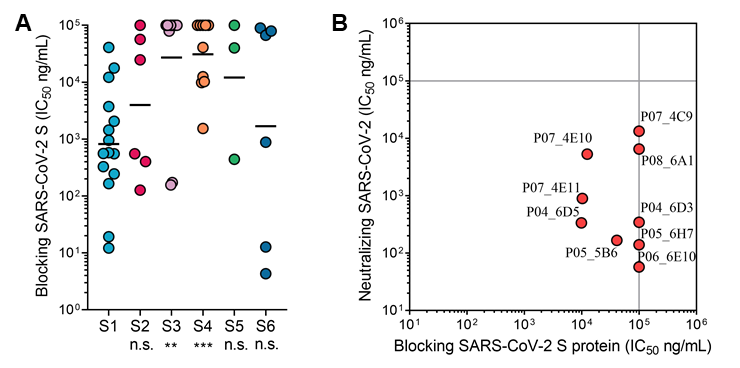
**

**Figure S13.**

**
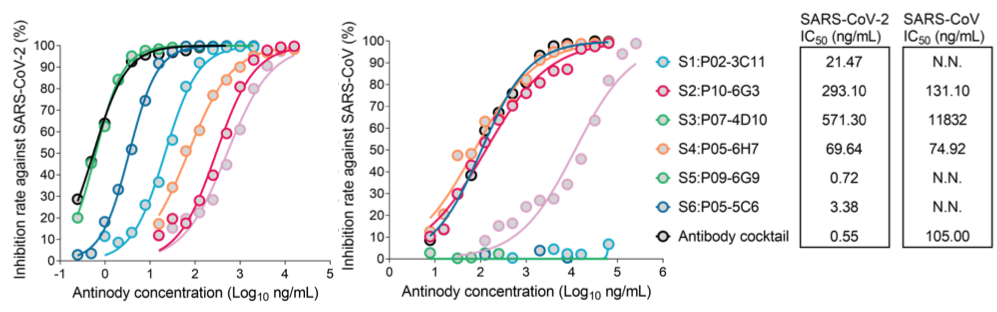
**

**Figure S14.**

**
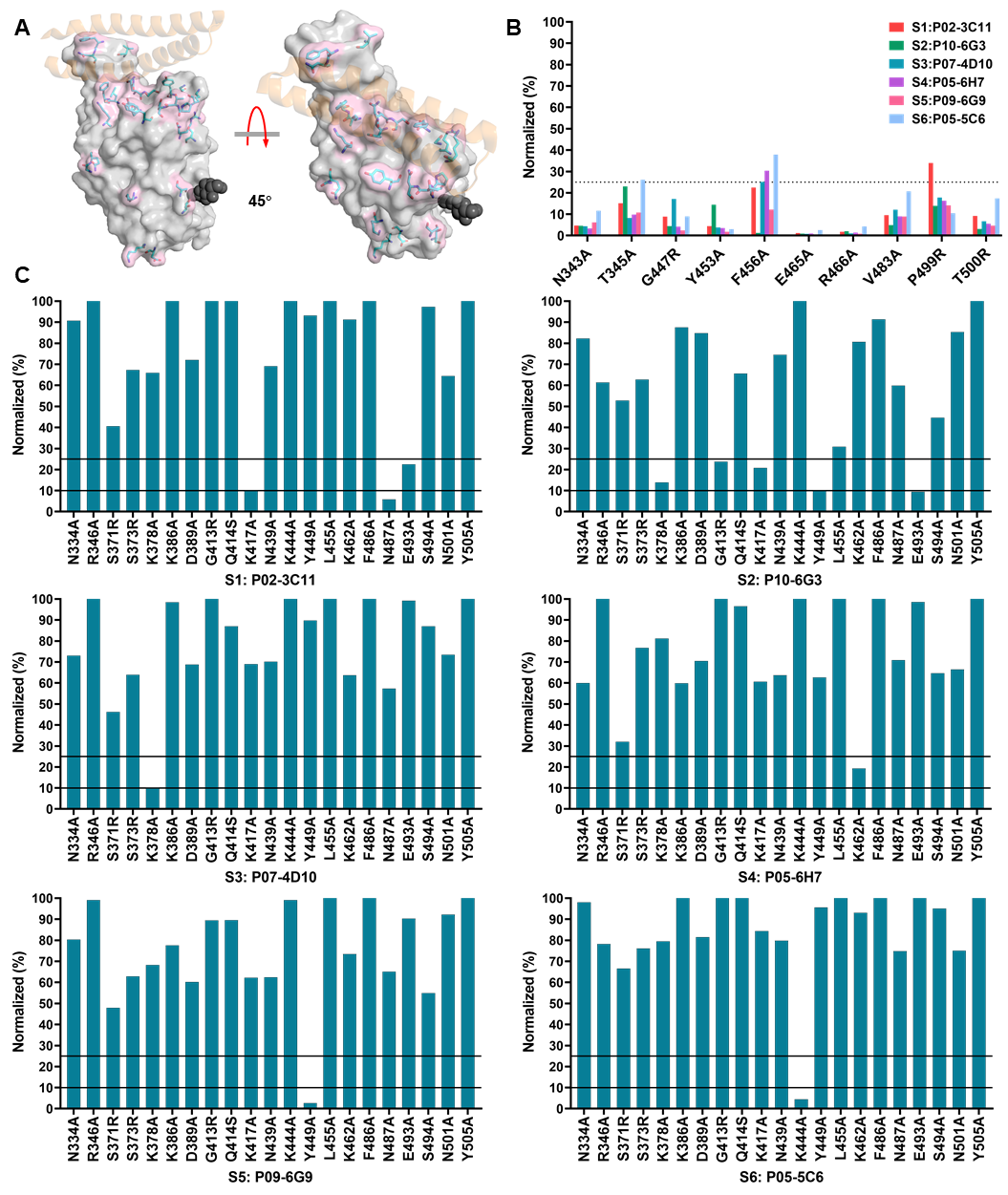
**

**Figure S15.**

**
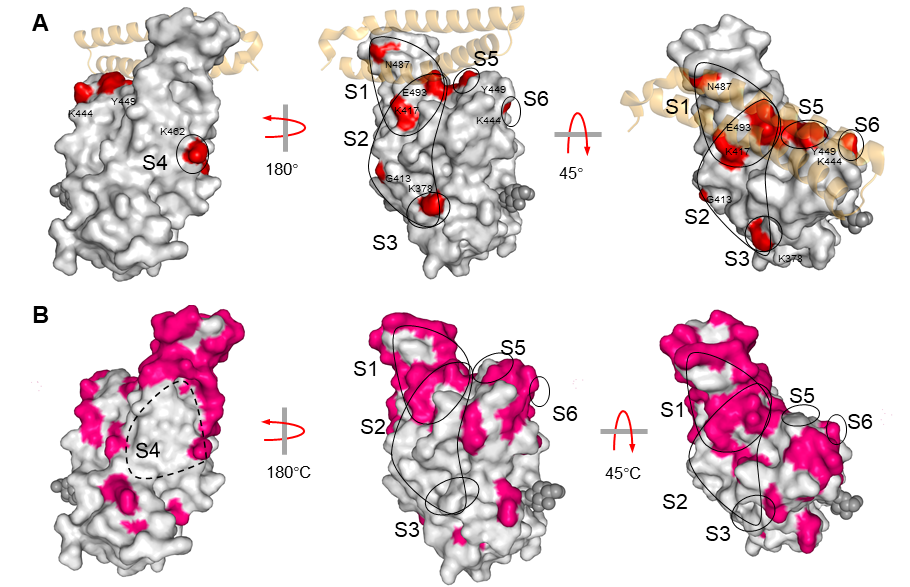
**

**Figure S16.**

**
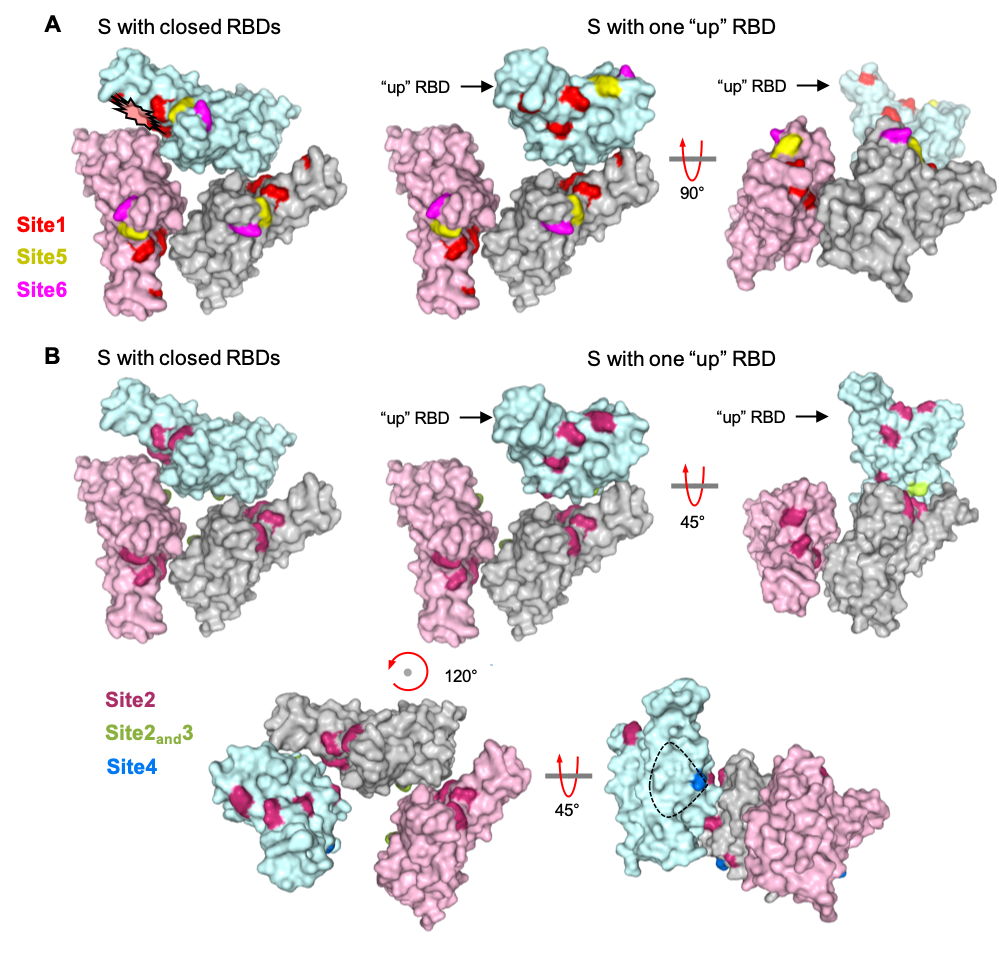
**

**Figure S17.**

**
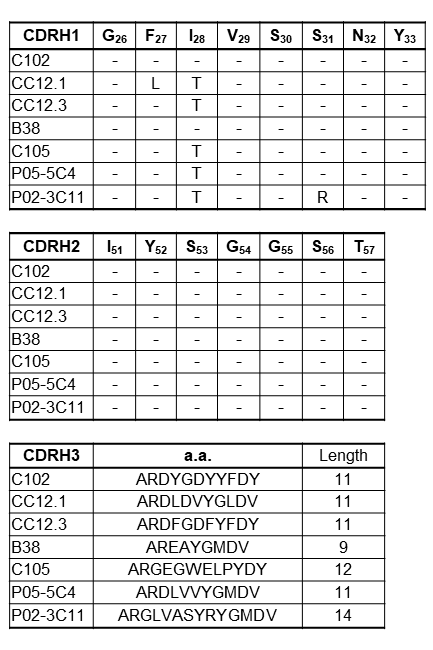
**

| **Table. S1. Information of COVID-19 convalescent individuals**. F: female M: male. | | | | | | | | | | | |
| --- | --- | --- | --- | --- | --- | --- | --- | --- | --- | --- | --- |
| ID | Age | Gender | Severity | Symptom onset date | Hospitalization date | PBMC collection date | First Symptoms | Chronic basic disease | Infection of other virus | CT | SARS-CoV-2 RNA |
| P01 | 54 | F | mild | 1.25 | 1.26-2.11 | 2.6 | Cough | hypertension | none | Bilateral | + |
| P02 | 64 | F | mild | 2.7 | 2.8-2.24 | 2.29 | Fever | none | none | Bilateral | + |
| P03 | 69 | M | mild | 2.9 | 2.9-2.25 | 2.29 | Fever | hypertension, hematencephalon | none | Bilateral | + |
| P04 | 33 | F | mild | 2.8 | 2.9-2.26 | 2.29 | Fever | none | HBV | unilateral | + |
| P05 | 40 | M | mild | 1.21 | 1.26-2.20 | 3.5 | Fever | none | none | Bilateral | + |
| P06 | 68 | M | mild | 1.28 | 1.30-2.20 | 3.6 | Fever | hypertension | none | Bilateral | + |
| P07 | 50 | F | mild | 1.3 | 2.4-2.20 | 3.7 | Fever, Cough | none | none | Bilateral | + |
| P08 | 47 | M | mild | 1.26 | 1.28-2.20 | 3.7 | Fever | none | HBV | Bilateral | + |
| P09 | 71 | M | mild | 2.5 | 2.7-2.23 | 3.9 | Fever, Cough | hypertension, diabetes | none | Bilateral | + |
| P10 | 44 | M | mild | 1.28 | 1.30-2.27 | 3.14 | Fever, Cough | diabetes | none | Bilateral | + |

| **Table. S2. Plasma anti-RBD antibody titers and neutralization capacity for COVID-19 convalescent individuals.** | | | | | |
| --- | --- | --- | --- | --- | --- |
| ID | Duration of immune response (days) | Anti-RBD antibodies (S/CO) | Anti-RBD IgG (S/CO) | Anti-RBD IgM (S/CO) | Neutralization (ED_50_) |
| P01 | 12 | 931.58 | 113.16 | 1685.71 | 1798 |
| P02 | 22 | 1584.21 | 889.47 | 324.76 | 1728 |
| P03 | 20 | 28.74 | 24.05 | 0.69 | 235.9 |
| P04 | 21 | 2247.37 | 51.89 | 2819.05 | 3185 |
| P05 | 44 | 484.21 | 634.21 | 128.57 | 505.5 |
| P06 | 38 | 1021.05 | 505.26 | 1447.62 | 1041 |
| P07 | 36 | 710.53 | 445.26 | 7152.38 | 2276 |
| P08 | 41 | 473.68 | 305.79 | 1.95 | 1215 |
| P09 | 33 | 552.6 | 272.1 | 1476.2 | 820 |
| P10 | 46 | 334.7 | 17.7 | 1761.9 | 1001 |

**Table. S3. Amino acid sequence of diverse SARS-CoV-2 RBD variants expressed.**

| **ID** | **Amino acid sequence** |
| --- | --- |
| RBD_Glycan420,475_ | RVQPTESIVRFPNITNLCPFGEVFNATRFASVYAWNRKRISNCVADYSVLYNSASFSTFKCYGVSPTKLNDLCFTNVYADSFVIRGDEVRQIAPGQTGKIANYTYKLPDDFTGCVIAWNSNNLDSKVGGNYNYLYRLFRKSNLKPFERDISTEIYQNGTTPCNGVEGFNCYFPLQSYGFQPTNGVGYQPYRVVVLSFELL |
| RBD_Glycan458,475_ | RVQPTESIVRFPNITNLCPFGEVFNATRFASVYAWNRKRISNCVADYSVLYNSASFSTFKCYGVSPTKLNDLCFTNVYADSFVIRGDEVRQIAPGQTGKIADYNYKLPDDFTGCVIAWNSNNLDSKVGGNYNYLYRLFRNSTLKPFERDISTEIYQNGTTPCNGVEGFNCYFPLQSYGFQPTNGVGYQPYRVVVLSFELL |
| RBD_Trucation455-491_ | RVQPTESIVRFPNITNLCPFGEVFNATRFASVYAWNRKRISNCVADYSVLYNSASFSTFKCYGVSPTKLNDLCFTNVYADSFVIRGDEVRQIAPGQTGKIADYNYKLPDDFTGCVIAWNSNNLDSKVGGNYNYLYRGGGGSLQSYGFQPTNGVGYQPYRVVVLSFELL |
| RBD_Trucation470-491_ | RVQPTESIVRFPNITNLCPFGEVFNATRFASVYAWNRKRISNCVADYSVLYNSASFSTFKCYGVSPTKLNDLCFTNVYADSFVIRGDEVRQIAPGQTGKIADYNYKLPDDFTGCVIAWNSNNLDSKVGGNYNYLYRLFRKSNLKPFERDISGGGGSLQSYGFQPTNGVGYQPYRVVVLSFELL |
| RBD-R346A | RVQPTESIVRFPNITNLCPFGEVFNATAFASVYAWNRKRISNCVADYSVLYNSASFSTFKCYGVSPTKLNDLCFTNVYADSFVIRGDEVRQIAPGQTGKIADYNYKLPDDFTGCVIAWNSNNLDSKVGGNYNYLYRLFRKSNLKPFERDISTEIYQAGSTPCNGVEGFNCYFPLQSYGFQPTNGVGYQPYRVVVLSFELL |
| RBD-S371R | RVQPTESIVRFPNITNLCPFGEVFNATRFASVYAWNRKRISNCVADYSVLYNRASFSTFKCYGVSPTKLNDLCFTNVYADSFVIRGDEVRQIAPGQTGKIADYNYKLPDDFTGCVIAWNSNNLDSKVGGNYNYLYRLFRKSNLKPFERDISTEIYQAGSTPCNGVEGFNCYFPLQSYGFQPTNGVGYQPYRVVVLSFELL |
| RBD-S373R | RVQPTESIVRFPNITNLCPFGEVFNATRFASVYAWNRKRISNCVADYSVLYNSARFSTFKCYGVSPTKLNDLCFTNVYADSFVIRGDEVRQIAPGQTGKIADYNYKLPDDFTGCVIAWNSNNLDSKVGGNYNYLYRLFRKSNLKPFERDISTEIYQAGSTPCNGVEGFNCYFPLQSYGFQPTNGVGYQPYRVVVLSFELL |
| RBD-K378A | RVQPTESIVRFPNITNLCPFGEVFNATRFASVYAWNRKRISNCVADYSVLYNSASFSTFACYGVSPTKLNDLCFTNVYADSFVIRGDEVRQIAPGQTGKIADYNYKLPDDFTGCVIAWNSNNLDSKVGGNYNYLYRLFRKSNLKPFERDISTEIYQAGSTPCNGVEGFNCYFPLQSYGFQPTNGVGYQPYRVVVLSFELL |
| RBD-K386A | RVQPTESIVRFPNITNLCPFGEVFNATRFASVYAWNRKRISNCVADYSVLYNSASFSTFKCYGVSPTALNDLCFTNVYADSFVIRGDEVRQIAPGQTGKIADYNYKLPDDFTGCVIAWNSNNLDSKVGGNYNYLYRLFRKSNLKPFERDISTEIYQAGSTPCNGVEGFNCYFPLQSYGFQPTNGVGYQPYRVVVLSFELL |
| RBD-D389A | RVQPTESIVRFPNITNLCPFGEVFNATRFASVYAWNRKRISNCVADYSVLYNSASFSTFKCYGVSPTKLNALCFTNVYADSFVIRGDEVRQIAPGQTGKIADYNYKLPDDFTGCVIAWNSNNLDSKVGGNYNYLYRLFRKSNLKPFERDISTEIYQAGSTPCNGVEGFNCYFPLQSYGFQPTNGVGYQPYRVVVLSFELL |
| RBD-G413R | RVQPTESIVRFPNITNLCPFGEVFNATRFASVYAWNRKRISNCVADYSVLYNSASFSTFKCYGVSPTKLNDLCFTNVYADSFVIRGDEVRQIAPRQTGKIADYNYKLPDDFTGCVIAWNSNNLDSKVGGNYNYLYRLFRKSNLKPFERDISTEIYQAGSTPCNGVEGFNCYFPLQSYGFQPTNGVGYQPYRVVVLSFELL |
| RBD-Q414S | RVQPTESIVRFPNITNLCPFGEVFNATRFASVYAWNRKRISNCVADYSVLYNSASFSTFKCYGVSPTKLNDLCFTNVYADSFVIRGDEVRQIAPGSTGKIADYNYKLPDDFTGCVIAWNSNNLDSKVGGNYNYLYRLFRKSNLKPFERDISTEIYQAGSTPCNGVEGFNCYFPLQSYGFQPTNGVGYQPYRVVVLSFELL |
| RBD-K417A | RVQPTESIVRFPNITNLCPFGEVFNATRFASVYAWNRKRISNCVADYSVLYNSASFSTFKCYGVSPTKLNDLCFTNVYADSFVIRGDEVRQIAPGQTGAIADYNYKLPDDFTGCVIAWNSNNLDSKVGGNYNYLYRLFRKSNLKPFERDISTEIYQAGSTPCNGVEGFNCYFPLQSYGFQPTNGVGYQPYRVVVLSFELL |
| RBD-N439A | RVQPTESIVRFPNITNLCPFGEVFNATRFASVYAWNRKRISNCVADYSVLYNSASFSTFKCYGVSPTKLNDLCFTNVYADSFVIRGDEVRQIAPGQTGKIADYNYKLPDDFTGCVIAWNSANLDSKVGGNYNYLYRLFRKSNLKPFERDISTEIYQAGSTPCNGVEGFNCYFPLQSYGFQPTNGVGYQPYRVVVLSFELL |
| RBD-K444A | RVQPTESIVRFPNITNLCPFGEVFNATRFASVYAWNRKRISNCVADYSVLYNSASFSTFKCYGVSPTKLNDLCFTNVYADSFVIRGDEVRQIAPGQTGKIADYNYKLPDDFTGCVIAWNSNNLDSAVGGNYNYLYRLFRKSNLKPFERDISTEIYQAGSTPCNGVEGFNCYFPLQSYGFQPTNGVGYQPYRVVVLSFELL |
| RBD-G447R | RVQPTESIVRFPNITNLCPFGEVFNATRFASVYAWNRKRISNCVADYSVLYNSASFSTFKCYGVSPTKLNDLCFTNVYADSFVIRGDEVRQIAPGQTGKIADYNYKLPDDFTGCVIAWNSNNLDSKVGRNYNYLYRLFRKSNLKPFERDISTEIYQAGSTPCNGVEGFNCYFPLQSYGFQPTNGVGYQPYRVVVLSFELL |
| RBD-Y449A | RVQPTESIVRFPNITNLCPFGEVFNATRFASVYAWNRKRISNCVADYSVLYNSASFSTFKCYGVSPTKLNDLCFTNVYADSFVIRGDEVRQIAPGQTGKIADYNYKLPDDFTGCVIAWNSNNLDSKVGGNANYLYRLFRKSNLKPFERDISTEIYQAGSTPCNGVEGFNCYFPLQSYGFQPTNGVGYQPYRVVVLSFELL |
| RBD-Y453A | RVQPTESIVRFPNITNLCPFGEVFNATRFASVYAWNRKRISNCVADYSVLYNSASFSTFKCYGVSPTKLNDLCFTNVYADSFVIRGDEVRQIAPGQTGKIADYNYKLPDDFTGCVIAWNSNNLDSKVGGNYNYLARLFRKSNLKPFERDISTEIYQAGSTPCNGVEGFNCYFPLQSYGFQPTNGVGYQPYRVVVLSFELL |
| RBD-L455A | RVQPTESIVRFPNITNLCPFGEVFNATRFASVYAWNRKRISNCVADYSVLYNSASFSTFKCYGVSPTKLNDLCFTNVYADSFVIRGDEVRQIAPGQTGKIADYNYKLPDDFTGCVIAWNSNNLDSKVGGNYNYLYRAFRKSNLKPFERDISTEIYQAGSTPCNGVEGFNCYFPLQSYGFQPTNGVGYQPYRVVVLSFELL |
| RBD-F456A | RVQPTESIVRFPNITNLCPFGEVFNATRFASVYAWNRKRISNCVADYSVLYNSASFSTFKCYGVSPTKLNDLCFTNVYADSFVIRGDEVRQIAPGQTGKIADYNYKLPDDFTGCVIAWNSNNLDSKVGGNYNYLYRLARKSNLKPFERDISTEIYQAGSTPCNGVEGFNCYFPLQSYGFQPTNGVGYQPYRVVVLSFELL |
| RBD-V483A | RVQPTESIVRFPNITNLCPFGEVFNATRFASVYAWNRKRISNCVADYSVLYNSASFSTFKCYGVSPTKLNDLCFTNVYADSFVIRGDEVRQIAPGQTGKIADYNYKLPDDFTGCVIAWNSNNLDSKVGGNYNYLYRLFRKSNLKPFERDISTEIYQAGSTPCNGAEGFNCYFPLQSYGFQPTNGVGYQPYRVVVLSFELL |
| RBD-F486A | RVQPTESIVRFPNITNLCPFGEVFNATRFASVYAWNRKRISNCVADYSVLYNSASFSTFKCYGVSPTKLNDLCFTNVYADSFVIRGDEVRQIAPGQTGKIADYNYKLPDDFTGCVIAWNSNNLDSKVGGNYNYLYRLFRKSNLKPFERDISTEIYQAGSTPCNGVEGANCYFPLQSYGFQPTNGVGYQPYRVVVLSFELL |
| RBD-N487A | RVQPTESIVRFPNITNLCPFGEVFNATRFASVYAWNRKRISNCVADYSVLYNSASFSTFKCYGVSPTKLNDLCFTNVYADSFVIRGDEVRQIAPGQTGKIADYNYKLPDDFTGCVIAWNSNNLDSKVGGNYNYLYRLFRKSNLKPFERDISTEIYQAGSTPCNGVEGFACYFPLQSYGFQPTNGVGYQPYRVVVLSFELL |
| RBD-Y489A | RVQPTESIVRFPNITNLCPFGEVFNATRFASVYAWNRKRISNCVADYSVLYNSASFSTFKCYGVSPTKLNDLCFTNVYADSFVIRGDEVRQIAPGQTGKIADYNYKLPDDFTGCVIAWNSNNLDSKVGGNYNYLYRLFRKSNLKPFERDISTEIYQAGSTPCNGVEGFNCAFPLQSYGFQPTNGVGYQPYRVVVLSFELL |
| RBD-Q493A | RVQPTESIVRFPNITNLCPFGEVFNATRFASVYAWNRKRISNCVADYSVLYNSASFSTFKCYGVSPTKLNDLCFTNVYADSFVIRGDEVRQIAPGQTGKIADYNYKLPDDFTGCVIAWNSNNLDSKVGGNYNYLYRLFRKSNLKPFERDISTEIYQAGSTPCNGVEGFNCYFPLASYGFQPTNGVGYQPYRVVVLSFELL |
| RBD-S494A | RVQPTESIVRFPNITNLCPFGEVFNATRFASVYAWNRKRISNCVADYSVLYNSASFSTFKCYGVSPTKLNDLCFTNVYADSFVIRGDEVRQIAPGQTGKIADYNYKLPDDFTGCVIAWNSNNLDSKVGGNYNYLYRLFRKSNLKPFERDISTEIYQAGSTPCNGVEGFNCYFPLQAYGFQPTNGVGYQPYRVVVLSFELL |
| RBD-P499R | RVQPTESIVRFPNITNLCPFGEVFNATRFASVYAWNRKRISNCVADYSVLYNSASFSTFKCYGVSPTKLNDLCFTNVYADSFVIRGDEVRQIAPGQTGKIADYNYKLPDDFTGCVIAWNSNNLDSKVGGNYNYLYRLFRKSNLKPFERDISTEIYQAGSTPCNGVEGFNCYFPLQSYGFQRTNGVGYQPYRVVVLSFELL |
| RBD-T500R | RVQPTESIVRFPNITNLCPFGEVFNATRFASVYAWNRKRISNCVADYSVLYNSASFSTFKCYGVSPTKLNDLCFTNVYADSFVIRGDEVRQIAPGQTGKIADYNYKLPDDFTGCVIAWNSNNLDSKVGGNYNYLYRLFRKSNLKPFERDISTEIYQAGSTPCNGVEGFNCYFPLQSYGFQPRNGVGYQPYRVVVLSFELL |
| RBD-N501A | RVQPTESIVRFPNITNLCPFGEVFNATRFASVYAWNRKRISNCVADYSVLYNSASFSTFKCYGVSPTKLNDLCFTNVYADSFVIRGDEVRQIAPGQTGKIADYNYKLPDDFTGCVIAWNSNNLDSKVGGNYNYLYRLFRKSNLKPFERDISTEIYQAGSTPCNGVEGFNCYFPLQSYGFQPTAGVGYQPYRVVVLSFELL |
| RBD-Y505A | RVQPTESIVRFPNITNLCPFGEVFNATRFASVYAWNRKRISNCVADYSVLYNSASFSTFKCYGVSPTKLNDLCFTNVYADSFVIRGDEVRQIAPGQTGKIADYNYKLPDDFTGCVIAWNSNNLDSKVGGNYNYLYRLFRKSNLKPFERDISTEIYQAGSTPCNGVEGFNCYFPLQSYGFQPTNGVGAQPYRVVVLSFELL |
| RBD-N343A | RVQPTESIVRFPNITNLCPFGEVFAATRFASVYAWNRKRISNCVADYSVLYNSASFSTFKCYGVSPTKLNDLCFTNVYADSFVIRGDEVRQIAPGQTGKIADYNYKLPDDFTGCVIAWNSNNLDSKVGGNYNYLYRLFRKSNLKPFERDISTEIYQAGSTPCNGVEGFNCYFPLQSYGFQPTNGVGYQPYRVVVLSFELL |
| RBD-T345A | RVQPTESIVRFPNITNLCPFGEVFNAARFASVYAWNRKRISNCVADYSVLYNSASFSTFKCYGVSPTKLNDLCFTNVYADSFVIRGDEVRQIAPGQTGKIADYNYKLPDDFTGCVIAWNSNNLDSKVGGNYNYLYRLFRKSNLKPFERDISTEIYQAGSTPCNGVEGFNCYFPLQSYGFQPTNGVGYQPYRVVVLSFELL |
| RBD-N334A | RVQPTESIVRFPNITALCPFGEVFNATRFASVYAWNRKRISNCVADYSVLYNSASFSTFKCYGVSPTKLNDLCFTNVYADSFVIRGDEVRQIAPGQTGKIADYNYKLPDDFTGCVIAWNSNNLDSKVGGNYNYLYRLFRKSNLKPFERDISTEIYQAGSTPCNGVEGFNCYFPLQSYGFQPTNGVGYQPYRVVVLSFELL |
| RBD-E340A | RVQPTESIVRFPNITNLCPFGAVFNATRFASVYAWNRKRISNCVADYSVLYNSASFSTFKCYGVSPTKLNDLCFTNVYADSFVIRGDEVRQIAPGQTGKIADYNYKLPDDFTGCVIAWNSNNLDSKVGGNYNYLYRLFRKSNLKPFERDISTEIYQAGSTPCNGVEGFNCYFPLQSYGFQPTNGVGYQPYRVVVLSFELL |
| RBD-R466A | RVQPTESIVRFPNITNLCPFGEVFNATRFASVYAWNRKRISNCVADYSVLYNSASFSTFKCYGVSPTKLNDLCFTNVYADSFVIRGDEVRQIAPGQTGKIADYNYKLPDDFTGCVIAWNSNNLDSKVGGNYNYLYRLFRKSNLKPFEADISTEIYQAGSTPCNGVEGFNCYFPLQSYGFQPTNGVGYQPYRVVVLSFELL |
| RBD-K462A | RVQPTESIVRFPNITNLCPFGEVFNATRFASVYAWNRKRISNCVADYSVLYNSASFSTFKCYGVSPTKLNDLCFTNVYADSFVIRGDEVRQIAPGQTGKIADYNYKLPDDFTGCVIAWNSNNLDSKVGGNYNYLYRLFRKSNLAPFERDISTEIYQAGSTPCNGVEGFNCYFPLQSYGFQPTNGVGYQPYRVVVLSFELL |
| RBD-E465A | VQPTESIVRFPNITNLCPFGEVFNATRFASVYAWNRKRISNCVADYSVLYNSASFSTFKCYGVSPTKLNDLCFTNVYADSFVIRGDEVRQIAPGQTGKIADYNYKLPDDFTGCVIAWNSNNLDSKVGGNYNYLYRLFRKSNLKPFARDISTEIYQAGSTPCNGVEGFNCYFPLQSYGFQPTNGVGYQPYRVVVLSFELL |
